# Exploitation of stable nanostructures based on the mouse polyomavirus for development of a recombinant vaccine against porcine circovirus 2

**DOI:** 10.1371/journal.pone.0184870

**Published:** 2017-09-18

**Authors:** Martin Fraiberk, Michaela Hájková, Magdaléna Krulová, Martina Kojzarová, Alena Drda Morávková, Ivan Pšikal, Jitka Forstová

**Affiliations:** 1 Charles University, Faculty of Science, Prague, Czech Republic; 2 Dyntec spol s r.o., Terezín, Czech Republic; Sun Yat-Sen University, CHINA

## Abstract

The aim of this study was to develop a suitable vaccine antigen against porcine circovirus 2 (PCV2), the causative agent of post-weaning multi-systemic wasting syndrome, which causes significant economic losses in swine breeding. Chimeric antigens containing PCV2b Cap protein sequences based on the mouse polyomavirus (MPyV) nanostructures were developed. First, universal vectors for baculovirus-directed production of chimeric MPyV VLPs or pentamers of the major capsid protein, VP1, were designed for their exploitation as vaccines against other pathogens. Various strategies were employed based on: A) exposure of selected immunogenic epitopes on the surface of MPyV VLPs by insertion into a surface loop of the VP1 protein, B) insertion of foreign protein molecules inside the VLPs, or C) fusion of a foreign protein or its part with the C-terminus of VP1 protein, to form giant pentamers of a chimeric protein. We evaluated these strategies by developing a recombinant vaccine against porcine circovirus 2. All candidate vaccines induced the production of antibodies against the capsid protein of porcine circovirus after immunization of mice. The candidate vaccine, Var C, based on fusion of mouse polyomavirus and porcine circovirus capsid proteins, could induce the production of antibodies with the highest PCV2 neutralizing capacity. Its ability to induce the production of neutralization antibodies was verified after immunization of pigs. The advantage of this vaccine, apart from its efficient production in insect cells and easy purification, is that it represents a DIVA (differentiating infected from vaccinated animals) vaccine, which also induces an immune response against the mouse polyoma VP1 protein and is thus able to distinguish between vaccinated and naturally infected animals.

## Introduction

Most antiviral vaccines are based on killed or attenuated viruses. These vaccines, although relatively safe, still represent a non-negligible risk of reversion to a virulent phenotype *in vivo*. VLPs (virus-like particles), which lack infectious genetic material, represent a modern and safe alternative to classical vaccines. Similarly to native virions, VLP nanostructures, due to their symmetry and repetitive structure, are able to cross-link surface immunoglobulins on the surface of B-cells, thus inducing a strong activation signal leading to B-cell proliferation and antibody production [1, 2]. They can serve as direct immunogens to stimulate humoral and cellular immune responses. Additionally, they are internalized by cells as efficiently as native virus particles and have comparable intracellular trafficking [3]. The capsid proteins of some viruses can form stable empty capsids structures spontaneously [4–6]. However, in the absence of viral nucleic acid, VLPs of some viruses are formed inefficiently and/or are unstable [7, 8]. Stable VLP structures and possibly other viral protein assemblies can serve as scaffolds for the preparation of chimeric nanostructures carrying foreign epitopes that may induce a similarly strong humoral response.

Various studies on the exploitation of VLPs of polyomaviruses, including MPyV VLPs as carriers of foreign epitopes, proteins and nucleic acids, are reviewed in Teunissen et al or in Suchanova et al [9,10]. Previously, we and others studied the potential of MPyV VLPs to carry DNA or proteins inside the MPyV VLPs [11–15].

Mouse polyomavirus is a small non-enveloped DNA virus with a circular 5.3-kbp genome that encodes three structural proteins, VP1, VP2 and VP3. The capsid shell with icosahedral symmetry is composed of 72 pentameric capsomeres of the major structural protein, VP1. Two minor capsid proteins, longer (VP2) and shorter (VP3) versions synthesized from the same open reading frame, are not exposed on the surface of the virion. Their common C-terminus interacts with the central cavity of VP1 pentamers and the N-terminus of either VP2 or VP3 and is oriented into the capsid interior. The structure of VP1 can be divided into three parts. The N-terminal part includes the nuclear localization signal (first 12 amino acids) and the DNA-binding domain, which interacts with DNA non-specifically [16,17]. The central part of the VP1 molecule is formed by α-helixes and β-sheets that are connected with loops. BC, DE and HI loops are exposed on the surface of the capsid. The C-terminal part of VP1 is very flexible and is responsible for inter-capsomeric contacts within the virus particle. VP1, the major capsid architecture protein, is responsible for recognition of the sialyzed ganglioside receptor on the cell surface. Importantly, VP1 can self-assemble spontaneously and efficiently into VLPs in the cell nucleus when expressed in mammalian, insect or yeast cells, or *in vitro* from VP1 pentamers produced in *E*. *coli*. [18–20]. VP2 and VP3 are not required for MPyV VLP formation. MPyV VLPs, as well as capsomeres (VP1 pentamers), are stable. They can be easily purified and, as the crystal structure of VP1 has been revealed, they can be easily modified [21].

In this study, we designed and prepared vectors for the development of chimeric vaccines based on stable VLPs and pentameric capsomeres of the mouse polyomavirus (MPyV) and tested the feasibility and immunogenic properties of MPyV-based chimeric structures by developing a vaccine against porcine circovirus 2 (PCV2).

Porcine circovirus type 2 (PCV2), first described in 1998 [22], is a non-enveloped single-stranded DNA virus and a member of the genus *Circovirus*, family *Circoviridae*. PCV2 exists in four genotypes, PCV2a, PCV2b, PCV2c and PCV2d [23]. While PCV2a was the dominant genotype in Europe until the year 2000. PCV2b has recently become prevalent [24]. PCV2c was identified in archived Danish tissues [25] of fetal pigs from Brazil [26] and PCV2d was identified in China and the USA [27, 28].

PCV2 is considered an infectious agent that causes several porcine circovirus-associated diseases (PCVAD). The clinical manifestation of PCVAD, post-weaning multi-systemic wasting syndrome (PMWS) [22], is characterized by chronic wasting and severely impaired weight gain in piglets 6 to 11 weeks old. The illness causes severe economic losses in swine industry. Although the presence of PCV2 is essential, only a few studies have been able to reproduce PMWS by inoculating with PCV2 alone [29]. Thus, another infectious or non-infectious factor must be present to develop PMWS [30]. The most successful method to reproduce the disease is coinfection with pathogens such porcine parvovirus (PPV) [31,32], porcine reproductive and respiratory syndrome virus (PRRSV) [33,34], torque teno virus TTV [35], *Mycoplasma hyopneumoniae* [36] or the use of immunostimulants, e.g. keyhole limpet hemocyanin in incomplete Freund’s adjuvant (KLH-ICFA) [37, 38].

PCV2 virions display icosahedral symmetry and a diameter of approximately 17 nm [39, 40]. They are assembled from a single capsid protein. The PCV2 genome is approx. 1.8 kb long and contains four open reading frames (ORFs). ORF1 encodes proteins Rep and Rep’, which are both necessary for replication [41]. The ORF3 product plays a role in apoptosis induction and is not essential for virus replication [42, 43]. ORF4 influences the function of ORF3 and antagonizes apoptosis [44]. ORF2 encodes one capsid protein (Cap)–the major antigenic determinant of the virus and the target for vaccine development. The capsid protein comprises three main areas that contain immunogenic epitopes and are targets of the humoral immune response [45]. Amino acids in positions 131, 151, 190 and the last three amino acids are involved in the conformation of neutralizing epitopes [46,47]. The non-protective immunogenic epitope, amino acid positions 169–180, is located inside the PCV2 capsid and appears to be a decoy epitope for the immune response. Pigs developing antibodies against this decoy epitope have significantly lower levels of neutralizing antibodies [48]. The crystal structure of PCV2 [49] allows the location of previously determined immunogenic epitopes in the context of the capsid. The Cap protein is able to assemble into VLP structures in insect [50] or yeast cells [51]; however, the efficiency of assembly is much lower than for MPyV VLPs. Moreover, self-assembled Cap VLPs were often less ordered than those in the purified PCV2 preparation (Nawagitgul et al [50] and our observations).

Here, we described the preparation of several variants of chimeric nanostructures based on MPyV VLP and capsomere scaffolds, carrying epitopes or the entire sequence of the PCV2 Cap protein. In addition, we compared the abilities of the nanostructures to induce Cap-specific humoral and cellular immune responses in mice. The candidate vaccines exhibiting the best results in the virus neutralization assay were further used for immunization of pigs and their immunogenic properties were compared to those of the Circoflex commercial vaccine.

## Material and methods

### Cells, viruses

Porcine kidney cells PK15 (ATCC CCL-33), kindly provided by the Dyntec company (www.dyntec.cz), were cultivated in Dulbecco’s modified Eagle’s medium (DMEM; Sigma) containing 5% FCS (fetal calf serum; Gibco) in a humidified incubator at 37°C in a 5% CO_2_ atmosphere. SF9 (*Spodoptera frugiperda)* insect cells (ThermoFisher Scientific) were cultivated as adherent cultures at 27°C in TNM-FH medium (Sigma) containing 10% FCS, supplemented with 4mM L-glutamine (Gibco). The inoculum (TCID_50_ = 10^5^) of PCV2b was kindly provided by the Dyntec company. PCV2b was originally identified in the Czech Republic by RT-PCR performed in the inguinal lymph node (sample L14181) of a pig with clinical manifestation of PMWS [52]. The sequence of the Cap gene is given in Supporting information S1 File.

### Construction of universal baculovirus transfer vectors for insertion of foreign sequences

**Fig 1:**
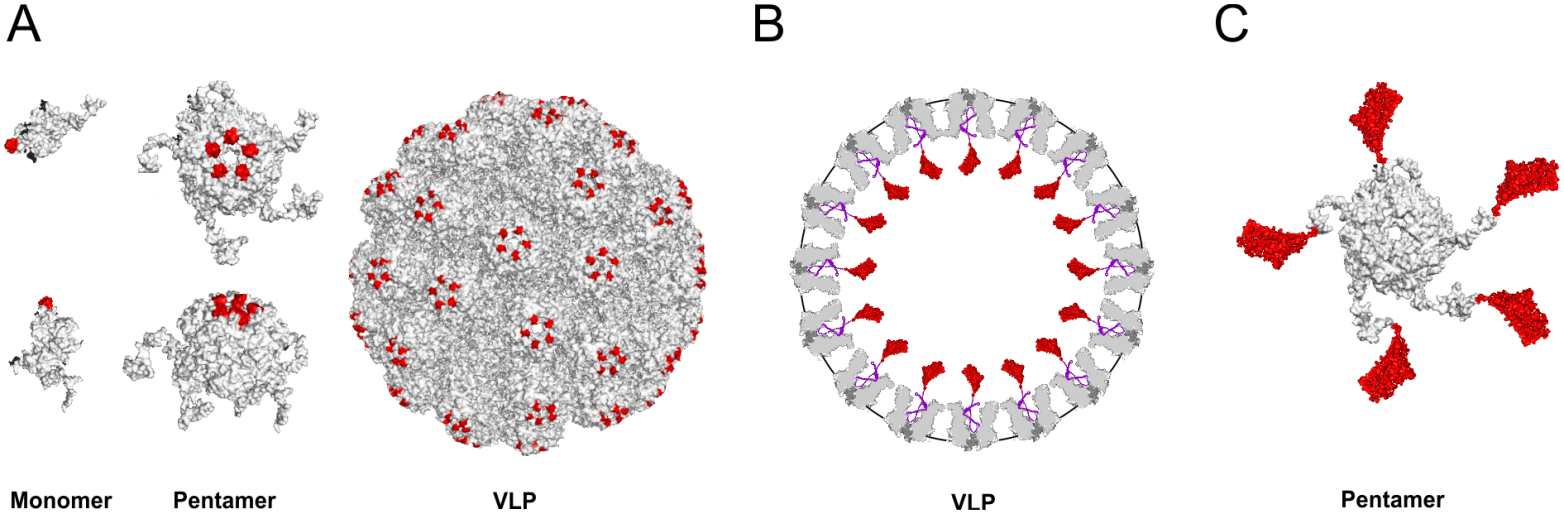
Design of chimeric structures based on MPyV capsid proteins, carrying sequences of PCV2- Cap. (**A**): Monomers, pentamers and VLPs of MPyV (grey) with immunogenic epitopes of PCV2 Cap (red) inserted into the DE loop of MPyV VP1 (VLP-A structure). (**B**): Entire PCV2 Cap (red) fused with truncated MPyV minor capsid protein—VP3 (violet) is situated inside the MPyV VLP (VLP-B structure). Cross-section of VLP (grey) is presented. (**C**): Entire PCV2 Cap (red) fused to the C-terminus of MPyV VP1 (grey) forming a pentameric capsomere (Capsomere C).

The transfer vector for production of VLP-A nanostructures (with foreign epitopes exposed on the VP1 VLP surface) is based on pFastBacI (ThermoFisher Scientific). First, the restriction site for BamHI was deleted from multiple cloning sites (MCS) of pFastBacI as follows: the transfer vector was digested with BamHI, overlapping ends blunted by the Klenow fragment of DNA polymerase I (ThermoFisher Scientific). The vector was circularized by T4 DNA ligase (ThermoFisher Scientific) and then reopened by EcoRI and KpnI. The MPyV VP1 gene was amplified in two parts (VP1a and VP1b). The pMJG plasmid [53] containing the entire MPyV genome served as a template. Primers for amplification were designed so that seven amino acids in the MPyV VP1 DE loop were deleted and replaced by a BamHI site surrounded by flexible glycine-serine (G-S)_3_ linkers. Manipulations to the DE loop had no effect on the stability of VP1 protein [54]. In addition, EcoRI and KpnI sites were added to the 5´end and 3´end of the VP1 gene, respectively. The modified fragments of the VP1 gene were connected through BamHI and inserted into the pFastBacI vector via EcoRI and KpnI restriction sites. The resulting construct pFastBacI-VP1_DEΔ7_ can serve as a universal cloning vector for insertions of sequences of different foreign immunogenic epitopes, through the BamHI restriction site, into the VP1 DE loop (Fig 1A).

The transfer vector for production of VLP-B nanostructures (with foreign protein or its parts enclosed inside VP1 VLPs), based on the pFastBacDual transfer vector (ThermoFisher Scientific) allowing expression of two foreign genes from two baculovirus promoters, was previously constructed in our laboratory. Briefly, the MPyV VP1 gene was inserted into the plasmid through EcoRI and XmaI under a polyhedrin promoter. The truncated MPyV VP3 gene (tVP3; last 3´end 99 amino acids) was amplified and inserted under the control of a baculovirus P_10_ promoter through the SmaI and NheI sites. Both MPyV gene sequences were amplified from the pMJG plasmid. The resulting construct pFastBacDual-VP1/tVP3 can serve as a universal cloning vector for fusion of sequences of foreign proteins with the 3´terminus of the tVP3 gene (Fig 1B). VLPs produced by the recombinant baculovirus prepared by this transfer vector contain a foreign protein in the interior, connected via VP3 sequences to the central cavities of pentameric VP1 capsomeres (Fig 1B).

For construction of a transfer vector for Capsomere-C nanostructures, the MPyV VP1 gene was amplified from the pMJG plasmid using primers designed to remove the stop codon and equip the gene with a BglII restriction site at the 5´end and BamHI and SalI restriction sites at the 3´end. VP1 sequences were introduced into the pFastBacI vector opened with BamHI and SalI (transfer vector pFastBac1-VP1). Sequences of a foreign protein can be inserted in frame into several restriction sites (e.g., BamHI, XbaI, PstI, KpnI, or HindIII). The recombinant baculovirus based on this transfer vector should express the VP1 protein fused at its flexible C-terminus with a foreign protein in the form of giant pentamers or higher complexes (Fig 1C).

Primers used for the construction of baculovirus transfer vectors are given in Supporting information S1 Table.

### Insertion of sequences of the capsid protein of porcine circovirus 2b (PCV2b) into universal baculovirus transfer vectors

**Fig 2:**
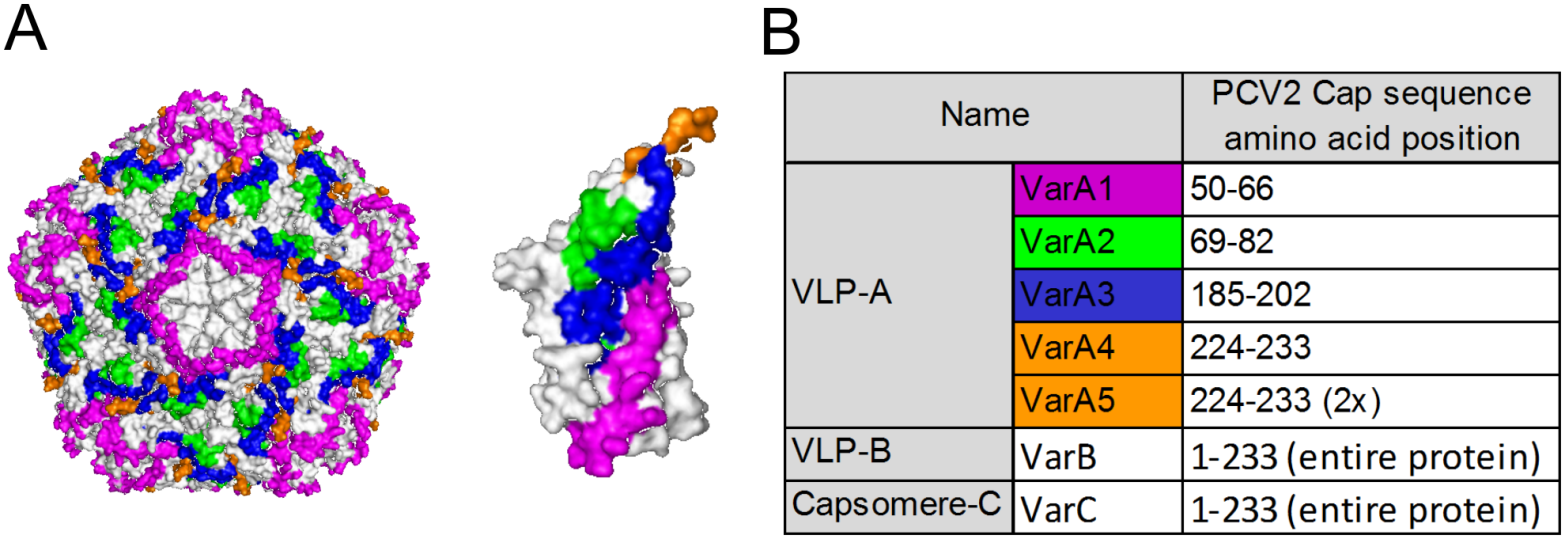
Model of PCV2 capsid. (**A**) Position of selected PCV2 Cap epitopes (colored) on the surface of PCV2 particle (left) or Cap monomer (right). (**B**) Table of Cap sequences used for construction of individual nanostructure variants.

For VLP-A (VarA1–A5) nanostructures, phosphorylated synthetic oligonucleotides (synthesized by the IDT company) encoding selected PCV2 Cap epitopes (Fig 2) were inserted via BamHI restriction sites into the pFastBacI-VP1_DEΔ7_ transfer vector. For VLP-B (VarB) structures, the entire PCV2 Cap gene was amplified by PCR using the bacterial pET28b-Cap-His plasmid carrying the Cap gene [52] as a template. The FLAG epitope sequence was introduced upstream of Cap via PCR primers. The FLAG-Cap sequence was fused in frame with tVP3 via SmaI and SacI restriction sites with pFastBacDual-VP1/tVP3. The resulting vector was termed pFastBacDual-VP1/FLAG-Cap-tVP3. For Capsomere-C (VarC) nanostructures, the entire PCV2b Cap sequence was amplified using PCR primers introducing BamHI sites to the 5´ end and a His-tag (6 x His) and KpnI to the 3´end; the sequence was cloned into the pFastBac1-VP1 vector cleaved by BamHI and KpnI. The resulting transfer vectors were termed pFastBac1-VP1-Cap-His. All transfer vectors were used to prepare of recombinant baculoviruses.

Phosphorylated oligonucleotides used for construction of baculovirus transfer vectors are given in Supporting information S2 Table.

### Recombinant baculovirus preparation

Recombinant baculoviruses were produced according to the manufacturer’s instructions (ThermoFisher Scientific, Bac to Bac system). Briefly, *E*. *coli* DH10Bac containing a bacmid and helper vector were transformed using individual transfer vectors. Recombinant bacmids from positive bacterial colonies were isolated (ThermoFisher Scientific, PureLink^™^ HiPure Plasmid DNA Miniprep Kit) and verified by PCR. SF9 insect cells were transfected with bacmids via lipofection (ThermoFisher Scientific, Celfectin II reagent). Recombinant baculoviruses released into the growth media were harvested 72 hours after transfection and used for further multiplications by infection of insect cells to generate high-titre viral stocks.

### SDS protein electrophoresis (SDS-PAGE) and Western blot analysis

Purified chimeric VLPs and nanostructures were boiled in Laemmli sample buffer for 5 minutes and resolved in 10% SDS-polyacrylamide gel [55]. Separated proteins were stained with Gelcode stain (ThermoFisher Scientific). For Western blot analysis, proteins were electro-transferred onto a nitrocellulose membrane (Serva) in cold blotting buffer (0.3% Tris, 1.44% glycine, 20% methanol) at 2.5 mA/cm^2^ for 3 hours. The membranes were incubated in 5% skim milk in PBS for 1 h. Immunostaining with primary and secondary antibodies was performed for 1 h and 40 min, respectively, and the membranes washed (3 x 10minutes) in PBS after each incubation. Membranes were developed using Pierce ECL Western Blotting Substrate reagent (ThermoFisher Scientific) and exposed to X-ray film (Agfa).

### Blue native polyacrylamide electrophoresis (BN-PAGE)

The method was adapted from Hornikova et al [56]. Briefly, purified nanostructures VarC were mixed with native PAGE sample buffer (ThermoFisher Scientific) and separated in a native 3–12% gradient gel. The separated complexes were visualized using Gelcode staining.

### Mass spectrometry

The bands of interest were cut from the gel and chopped into 1x1x1 mm pieces. The pieces were destained, and DTT and iodoacetamide were applied to reduce and block cysteines. The samples were trypsinized as described previously [57]. The dried-droplet method of sample preparation was employed and spectra were acquired in a 4800 Plus MALDI TOF/TOF analyzer (AB Sciex). Data were analyzed using an in-house running Mascot server 2.2.07 and matched against a database composed of the following background databases: baculovirus expression system, insect SF9 cell proteome database of common contaminants, and MPyV VP1 sequences. Cysteine carbamidomethylation was set as a fixed modification, and methionine oxidation and N, Q deamination were set as variable modifications. One missed cleavage site was allowed. Precursor accuracy was set to 50 ppm and the accuracy for MS/MS spectra to 0.25 Da.

### Immunofluorescence

PK15 cells grown on coverslips in 24-well plates were fixed with 3% formaldehyde for 30 minutes. Cells were permeabilized by 5-minute incubation in 0.1% Triton X-100 in PBS and washed (three times, 10 minutes) with PBS. Free epitopes were blocked by 1-hour incubation in 1% BSA in PBS. Cells were immunostained by 1-hour incubation in primary antibody, washed (three times, 10 minutes) with PBS and incubated (30 minutes) with secondary antibody. After washing in PBS, the coverslips with cells were briefly washed in deionized water, dried and mounted in DAPI Gold solution (ThermoFisher Scientific).

### Immunization of mice and pigs

Mice were divided into groups (5 mice/group) and animals in each group were immunized subcutaneously with three doses (in two-week intervals) of one of the prepared nanostructures. The amount of protein used for all three immunizations was 50 μg per dose (approx. 3.1 μg per 1 g of mouse at the time of first immunization). Fourteen days after the last immunization, mice were bled and sacrificed under total anesthesia with Halothane (Sigma). Six-week-old pigs were divided into three groups. A group of seven pigs was immunized by the VarC nanostructure, a group of three pigs by a commercial vaccine Circoflex (Boehringer-Ingelheim), and the control group of three pigs received adjuvants in PBS. Immunization was performed twice in a 3-week interval into the cervical muscle. A single 1-ml dose contained 100 μg of antigen (approx. 20 μg per 1 kg of pig at the time of the first immunization) and 30% of Polygen adjuvants (MVP Technologies). Immunization with the commercial vaccine Circoflex was performed according to the manufacturer’s instructions. Pigs were euthanized using a slaughter gun with subsequent exsanguination.

Before pig immunization, the candidate vaccine, VarC, was inactivated by binary ethylenimine prepared according to Rueda et al [58].

### ELISA analyses

The PCV2b virus was propagated in PK15 cells. The cells were infected with multiplicity MOI = 0.01 and 6 days after infection. The cells, including growth medium, were harvested and subjected to three-fold freezing and melting. Cell lysate was clarified by centrifugation (15000×g, 10 minutes, 4°C) and the virus was concentrated by ultracentrifugation at 25,000 rpm, 3 hours, 4°C, Beckman rotor SW28. Sediment was resuspended in cold PBS and protein concentration was measured by the Bradford assay [59]. ELISA 96-well plates (Nunc) containing 25 μg of crude PCV2 virus in 50 μl of PBS were incubated overnight at 4°C. The plates were washed five times with PBS containing 0.05% Tween-20 (Sigma) and blocked by 2-hour incubation in 2% skim milk in PBS. Then, 100 μl of serum (diluted as indicated) of control or immunized mice was applied to the wells and incubated for 1 hour. Anti PCV2 Cap monoclonal antibody was used as a positive control. After washing with PBS containing 0.05% Tween-20, 50 μl horseradish peroxidase (HRP)-conjugated secondary antibody was added for 1 hour. Wells were washed with PBS/Tween-20 (0.05%) and overlaid with 100 μl of substrate solution (ABTS, Sigma). The absorbance of each well was determined at 415 nm using a microplate reader (Epoch, BioTec Instruments).

The ELISA commercial set INgezim CIRCO IgG (Ingenasa) was used according to the manufacturer's instructions for measurement of anti PCV2 antibody development in immunized pigs.

### Electron microscopy

Parlodion-carbon-coated grids were applied on top of a 5-μl drop of sample and left to absorb the sample for 5 min. The grids were rinsed twice in a drop of filtered distilled water for 30 seconds and negatively stained twice by one-minute incubation on a drop of 2% phosphotungstic acid (PTA, pH 7.3, Sigma), left for 1 min and dried. Electron micrographs were recorded in a JEM-1011 electron microscope (JEOL) operating at 80 kV.

### Antibodies

The following primary antibodies were used in this study: mouse monoclonal antibody against MPyV VP1 [7] (ELISA dilution– 1:500), mouse monoclonal antibody against FLAG (Sigma, cat. no. F1804, WB dilution– 1:400), mouse monoclonal antibody against PCV2 Cap (Median Diagnostics, cat. no. 9051_Anti-PCV2 NC, IF and ELISA dilution 1:500) and mouse monoclonal antibody Penta His (Qiagen, cat. no. 34660, WB dilution– 1:400) against Histag. The following secondary antibodies were used: goat antibody against mouse immunoglobulins conjugated with HRP (Bio-Rad, cat. no. 5178–2504, WB dilution– 1:1000) and with Alexa Fluor-488 (ThermoFisher Scientific, cat. no. A32723, IF dilution 1:1000) and goat antibody against swine immunoglobulins conjugated with HRP (Santa Cruz Biotechnology, cat. no. sc-2463, WB dilution– 1:1000).

### Animals

Charles River BALB/c female mice at eight weeks were purchased from the breeding unit of the Institute of Molecular Genetics, CAS, Prague, or Velaz company, Prague. Pigs (MeLiM strain) at six weeks were purchased from the Institute of Animal Physiology and Genetics CAS, Libechov. The use of animals was approved by the local Animal Ethics Committee (Departmental committee of the Ministry of Education, Youth and Sports for Animal care n. 25 066/2011-30 and Departmental committee of Czech Academy of Science n. 32/2015).

### Isolation and quantitative determination of PCV2 DNA

Total genomic DNA was extracted from 200 μl of each serum sample using a NucleoSpin Blood isolation kit (Macherey-Nagel) according to the manufacturer's instructions.

Quantification of PCV2 DNA levels was performed using real-time PCR in a Step One Plus instrument (Applied Biotechnologies) in the TaqMan format as previously described by Brunborg et al [60]. In short, the TaqMan probe was labelled with 5'-FAM and 3'-BHQ1 fluorophores (Generi Biotech) and the primers were designed to amplify a 100-bp DNA fragment within the selected nucleotide sequence of the PCV2 Cap gene. Absolute quantification of PCV2 DNA was performed using calibration curves generated by means of external standard DNA obtained by cloning the PCV2 cap gene in a pCR 2.1 vector (ThermoFisher Scientific). Standard curves for PCV2 DNA quantification were generated using tenfold dilution of the linearized plasmids in the range of 8 log10.

### Serum neutralization assay

PCV2-neutralizing antibodies were detected using the serum neutralization assay adapted from Meerts et al [61] and Lefebre et al [62]. Porcine PK15 kidney cells were seeded into a 24-well plate at density of 1.5 ×10^5^/well. Sera originating from groups of animals (mice or pigs) immunized with the same nanostructure were mixed at equal volume and inactivated at 56°C for 1 hour. Then, 1 ml of PCV2 (TCID_50_ = 10^5^) in DMEM medium containing 5% FCS was added to individual serum mixtures and incubated for 1 hour at 37°C to allow antibodies to attach to the virus. The mixtures were used to infect PK15 cells growing on coverslips. After 1 hour of incubation at 37°C, the mixtures were removed, and the cells were washed with medium without serum and incubated with complete DMEM medium (5% FCS) for 36 hours. After fixation, an indirect immunofluorescence assay was performed using primary antibody against PCV2 Cap and Alexa Fluor-488 secondary antibody. The numbers of infected cells in the samples were calculated and compared to the number of infected cells obtained with controls (cells infected with virus mixed with sera from control group animals).

### Evaluation of cell immune responses

#### Lymphocyte stimulation

A single-cell suspension of spleen cells from BALB/c mice was prepared in RPMI 1640 medium (Sigma) containing 10% fetal calf serum (FCS, Sigma), antibiotics (100 u/ml of penicillin, 100 μg/ml streptomycin), 10mM HEPES buffer and 5×10^-5^M 2-mercaptoethanol (hereafter referred to as complete RPMI 1640 medium). The cells (75×10^5^ cells/ml) were cultured in a volume of 1 ml of complete RPMI 1640 medium in 24-well tissue culture plates (Nunc) unstimulated or stimulated with 50 μg/ml of the nanostructures VarA4, VarB, VarC and VP1_Con_ for a 48-h incubation period.

#### Detection of activation markers

To characterize the capacity of the nanostructures VarA4, VarB and VarC to trigger immune activation, cultured spleen cells were harvested and washed with PBS containing 0.5% bovine serum albumin (BSA) and centrifuged (250 × g, 8 min). Cells were stained for 30 min on ice with the following monoclonal antibodies (mAbs): fluorescein isothiocyanate (FITC)-labelled anti-CD4 antibody (clone GK1.5, BD Pharmingen), phycoerythrin (PE)-labelled anti-CD4 antibody (clone GK1.5, BD Pharmingen), allophycocyanin (APC)-labelled anti-CD8a antibody (clone 53–6.7, BioLegend), FITC-labelled anti-CD25 antibody (clone PC61, eBioscience), APC-labelled anti-CD25 antibody (clone PC61, BioLegend), Alexa Fluor 700-labelled anti-CD45 antibody (clone 30-F11, BioLegend) and PE-labelled anti-CD69 antibody (clone H1.2F3, BioLegend). Dead cells were stained using Hoechst 33258 dye (Sigma) added to samples 15 min before flow cytometry analysis. Data were collected using a LSRII flow cytometer (BD Biosciences) and analyzed using Gatelogic 400.2A software (Invai).

#### Intracellular staining of cytokines

To evaluate the ratio of cytokine-producing lymphocytes, spleen cells were cultivated in the absence or presence of nanostructures VarA4, VarB, VarC and VP1 control (VP1_Con_) (50 μg/ml), and PMA (20 ng/ml Sigma), ionomycin (500 ng/ml, Sigma) and brefeldin A (5 μg/ml, eBioscience) were added to the cultures for the last 5 h of the 48-h incubation period. The cells were harvested and washed with PBS containing 0.5% BSA. Before intracellular staining, the cells were incubated for 30 min on ice with Alexa Fluor 700-labelled anti-CD45 antibody (clone 30-F11, BioLegend) and a Live/Dead Fixable Violet Dead Cell Stain Kit (Molecular probes, Eugene, OR) to stain dead cells. For intracellular staining, the cells were fixed and permeabilized using a Fixation/permeabilization Buffer Staining Kit (eBioscience) according to the manufacturer’s instructions. For detection of cytokine-producing lymphocytes, the cells were stained with the following mAbs: FITC-labelled anti-IFNγ antibody (clone, XMG1.2, BioLegend) and PE-labelled anti IL-4 antibody (clone 11B11, eBiosciences) for 30 min at 25°C. Data were collected using a LSRII flow cytometer (BD Biosciences) and analyzed using Gatelogic 400.2A software.

### Statistics

All results are expressed as means ± SD, or as SEM. The statistical significance of differences between the means of individual groups was calculated using one-way analysis of variance (ANOVA). A value of P≤ 0.05 was considered statistically significant.

## Results and discussion

### Design and preparation of universal vectors for chimeric nanostructure production

Three types of chimeric structures based on structural proteins of the mouse polyomavirus carrying foreign epitopes or entire proteins were designed (Fig 1): A) MPyV virus-like particles (VLPs), composed of MPyV major capsid protein, VP1, carrying a foreign epitope on their surface (VLP-A), B) MPyV VLPs carrying sequences of a foreign protein inside the particle (VLP-B) and C) MPyV pentameric capsomeres composed of pentamers of VP1 fused at its C-terminus with foreign protein sequences (Capsomere C). For production of chimeric structures, universal baculovirus transfer vectors were prepared (for a detailed description, see Material and methods). Briefly, for production of VLP-A structures, sequences of the top seven amino acids of the DE loop of MPyV VP1 were replaced using a cloning site for the insertion of a foreign epitope surrounded by sequences of a glycine-serine linker (pFastBac1 VP1_DEΔ7_). For production of VLP-B structures, a transfer vector ensuring expression of the VP1 gene (for VLP formation) and the C-terminal part of VP3 (responsible for interaction of MPyV minor capsid proteins with the central cavity of VP1 capsomere) was prepared. Cloning sites prior to the truncated VP3 gene sequences allow fusion of foreign gene sequences to sequence of truncated VP3. The fused protein should be situated inside VLPs (pFastBacDual VP1/tVP3). Finally, a baculovirus transfer vector (pFastBac1 VP1) for production of the Capsomere C structure carries the VP1 gene with an eliminated stop codon and several restriction sites for fusion with the N-terminus of a foreign gene. Fusion of foreign sequences to the 3´terminus of VP1 gene prevents VLP formation, oligomerization into pentameric VP1 capsomeres should not be affected.

The universal vectors were used for construction of baculoviruses producing candidate chimeric antigens for the development of vaccines against porcine circovirus 2.

### Preparation and characterization of nanostructures carrying the capsid protein of porcine circovirus 2 or its epitopes

First, we prepared chimeric MPyV VLPs carrying selected immunogenic epitopes from PCV2 Cap protein on their surface (VLP-A). For this, we synthetized four epitopes designed according to those previously described by Lekcharoensuk et al [45] and refined by Shang et al [47]. Lekcharoensuk et al [45] mapped conformational epitopes of the PCV2 capsid protein by analyses of PCV1-PCV2 ORF2 (open reading frame 2) chimeras in the context of the non-pathogenic PCV1 infectious genome using seven PCV2 monoclonal antibodies recognizing conformational epitopes. We selected four linear sequences and refined them according to their accessibility on the capsid surface using PCV2 capsid structure determination [42]. The selected epitopes (1–4) are shown in four different colors on the model of the PCV2 capsid (Fig 2A) and Cap monomer (Fig 2B) were inserted into the baculovirus transfer vector pFastBac1 VP1_DEΔ7_. Insertions of epitope sequences into the DE loop of MPyV VP1 were confirmed by sequencing. The fourth (yellow) epitope sequence (see Fig 2) was inserted into the transfer vector as a monomer (4) or dimer (5). From all five constructs, recombinant baculoviruses were prepared for production of VLPs carrying different epitopes (VarA1 –VarA5) in insect cells.

Furthermore, entire Cap protein-coding sequences (provided by the FLAG tag connected with 5’terminus of the Cap gene) were fused with tVP3 in the pFastBacDual VP1/tVP3 transfer vector, and recombinant baculovirus was prepared for production of VLPs with Cap sequences inside VLPs (VarB). We did not expect a high level of Cap-specific humoral response induced by this nanostructure but were interested in whether this nanostructure could induce cellular responses. A previous study by Tegerstedt et al [15] showed that MPyV-VLPs carrying inside a fusion protein between MPyV-VP2 and the extracellular and transmembrane domains of human Her2/neu (a proto-oncogene overexpressed in many epithelial carcinomas) induced a rejection response against a tumor inoculum and inhibition of spontaneous tumor outgrowth in a mutant Her2 transgenic mouse model.

Finally, the cap gene connected at its 3’end with His tag sequences was fused with the 3’end of VP1 gene in the pFastBac1 VP1 transfer vector, and recombinant baculovirus for production of chimeric VP1 capsomeres (VarC) was prepared. FLAG (in VarB) and His tag (in VarC) were inserted for detection of Cap fusion proteins by immune analysis (antibody against the Cap protein for western blot analyses is not available) and for isolation of VarC nanostructures.

**Fig 3:**
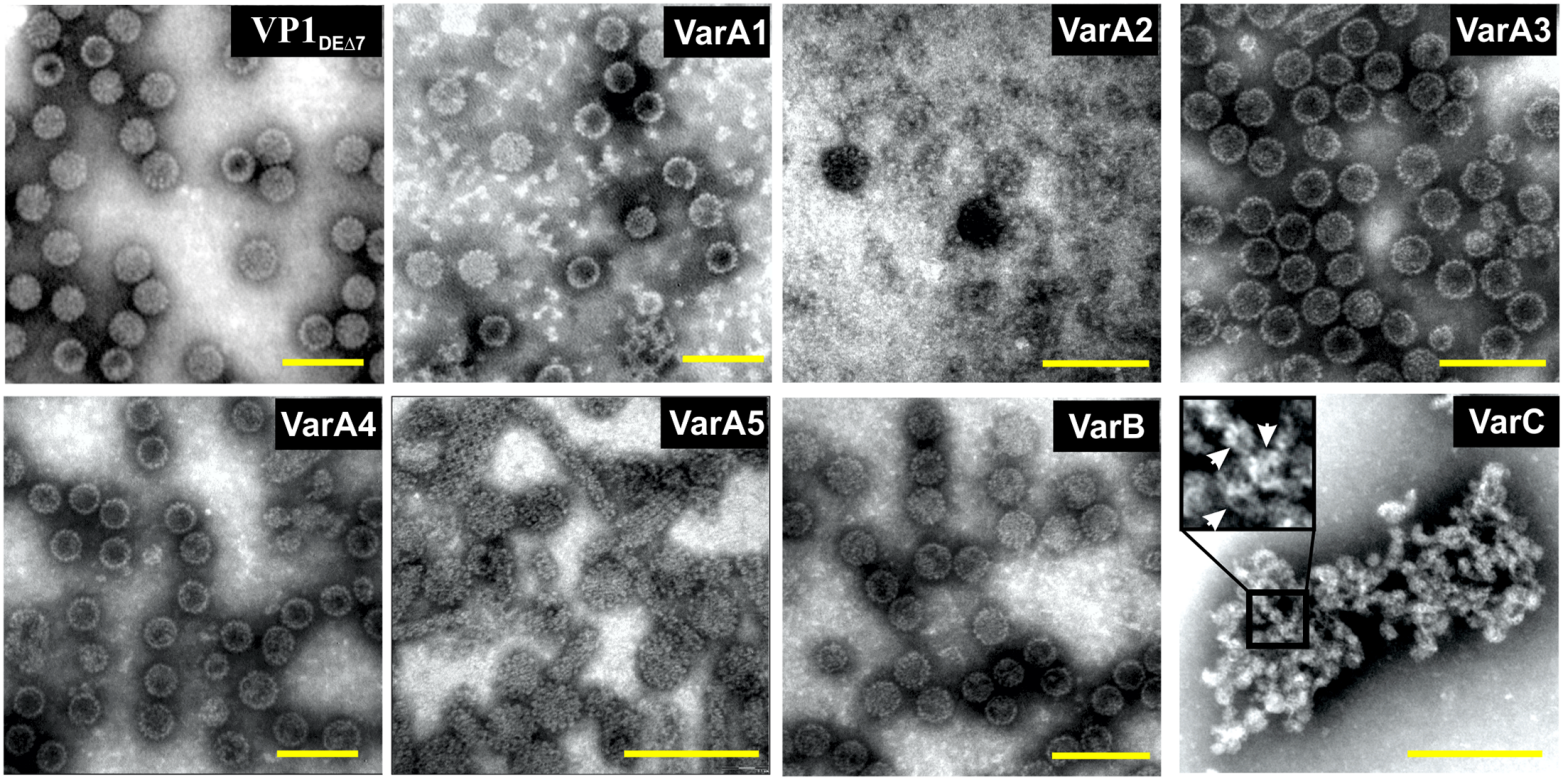
Electron microscopy of isolated structures. Negative staining with 2% PTA. Bars represent 100 nm.

Lysates of insect cells infected with individual recombinant baculoviruses were used for isolation of chimeric structures. Yields of isolated chimeric nanostructures ranged from 48 mg/L to 80 mg/L. The presence of epitopes inserted in the DE loop region of all five variants was verified by mass spectroscopy. Electron microscopy (EM) revealed that VarA1, VarA3 and VarA4 of chimeric VP1 protein efficiently formed VLP structures, similar to VP1_DEΔ7_ (Fig 3). However, production of VP1 VarA2 did not yield stable VLPs. The EM picture of VarA2 showed pentamers of non-assembled VLPs or pentamers complexed into irregular higher assemblies. VP1 VarA5 is assembled into VLPs and into filamentous structures of different diameters. VLPs were observed in the image of VarB designed to carry the Cap protein inside. VarC formed VP1-Cap fusion protein complexes with regular morphology, reminiscent of giant VP1 pentamers by their central hole. These structures formed large aggregates. Apart from the ability of MPyV VP1 to form stable pentamers, the Cap-Cap interaction can apparently cause further complexing of the fusion protein structures. The Cap protein was shown to produce stable dimers [63].

**Fig 4:**
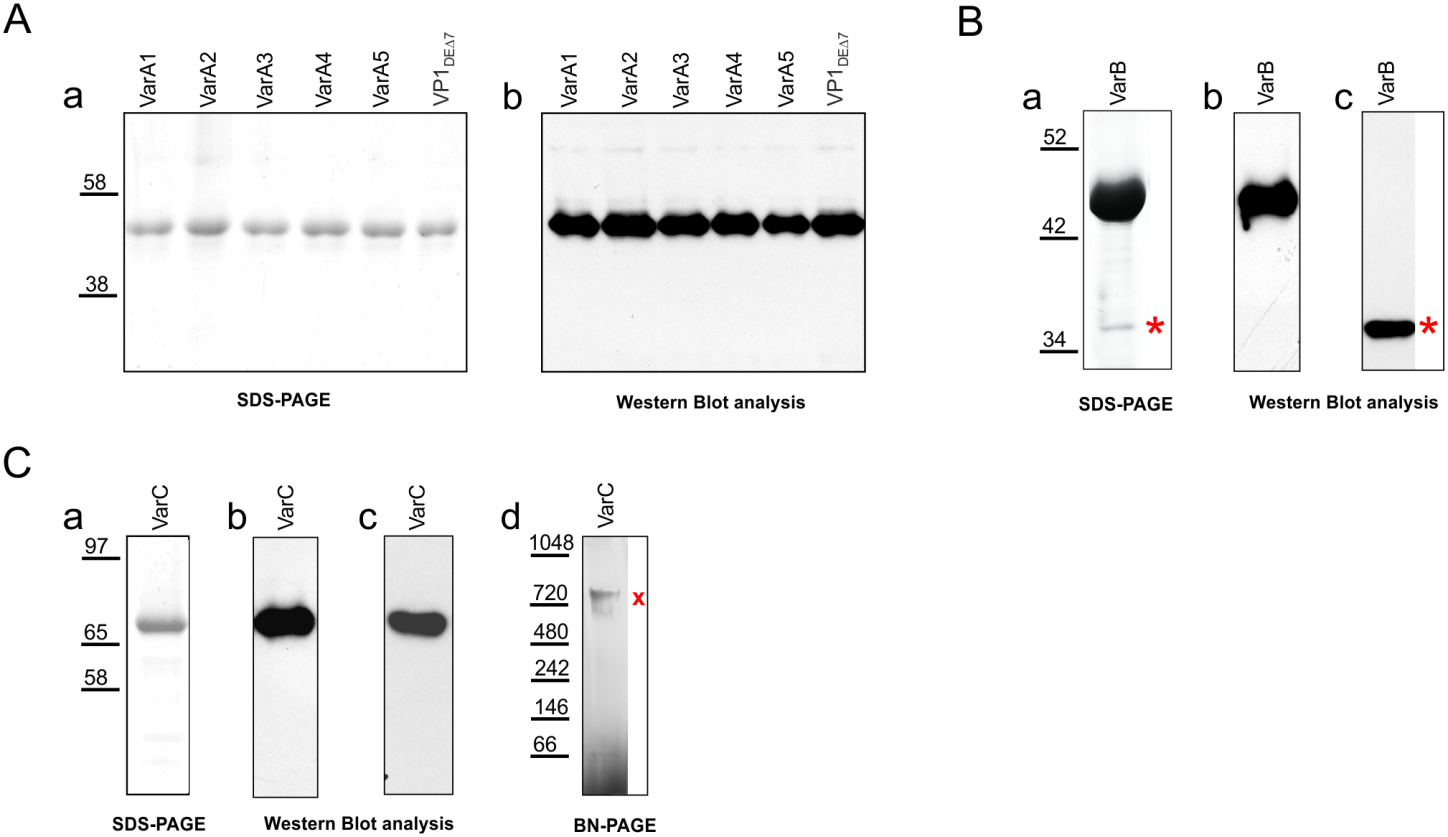
SDS PAGE and Western blot analyses of purified structures. Lysates of isolated structures resolved by SDS PAGE followed by Coomassie brilliant blue staining (**a**), Western blot analyses (**b, c**). Lysate of VarC resolved on Blue native electrophoresis stained by Gelcode stain. (**d**). (**A**) VLP-A (Var A1 –A5) and control VLPs composed of VP1_DEΔ7_. (**B**) VLP-B (VarB). (**C**) Capsomere C (VarC). Antibodies used: antibody against VP1 (**Ab, Bb, Cb**), antibody against FLAG (**Bc**) and antibody against His tag (**Cc**). *mark: Cap-tVP3 band (**Ba, c**), **x** mark: band corresponding to double pentamer of VP1-Cap-His (**Cd**).

Isolated structures were further examined by SDS PAGE and Western blot analysis (Fig 4). Gelcode-stained SDS PAGE (Fig 4Aa) revealed that all variants of the VP1 protein with Cap epitopes inserted into the DE loop (VarA1 –VarA5) were stable and their mobilities corresponded approximately to the mobility of VP1 (45 kDa). No degraded VP1 was observed on Western blots stained with VP1 antibody (Fig 4Ab). Fig 4B showed SDS PAGE (a) and Western blot analyses (b,c) of the VarB structure. Additionally, a band corresponding to VP1 protein (Fig 4Ba and 4Bb), a band with mobility corresponding to the FLAG-Cap-tVP3 fusion protein (37 kDa) and interacting with antibody against FLAG was detected in the lysate of purified VLPs VarB (Fig 4Bc). In theory, a maximum of 72 molecules of FLAG-Cap-tVP3 can be incorporated into one particle (one molecule per central cavity of each VP1 capsomere). For rough estimation of the number of fused Cap molecules incorporated into one VLP, the densities of VP1 and FLAG-Cap-tVP3 bands of purified VLPs separated in SDS-PAGE and stained with Coomassie dye were measured. The estimate was 12 FLAG-Cap-tVP3 molecules per one particle. Analysis of the VP1-Cap fusion protein (VarC) is presented in Fig 4C. The band with expected mobility for the fusion protein (71 kDa) was detected on SDS-PAGE (Fig 4Ca) and its identity proved by antibodies against VP1 (Fig 4Cb) and His tag (Fig 4Cc). BN-PAGE (blue native electrophoresis) was employed to reveal high-molecular complexes of VarC (Fig 4Cd). As MPyV VP1 forms immediately after the translation of pentamers, we expected to see, among others, pentameric complexes. The molecular weight of the VP1-Cap-HIS pentamer is 356 kDa. Surprisingly, the only band detected in the PAGE gel corresponded to the mobility of a decamer (2x pentamer) of the fused VP1-Cap protein. A substantial portion of the loaded material did not enter the gel, confirming high aggregate formation.

### Immunization by chimeric nanostructures induced specific antibody responses in mice

**Fig 5:**
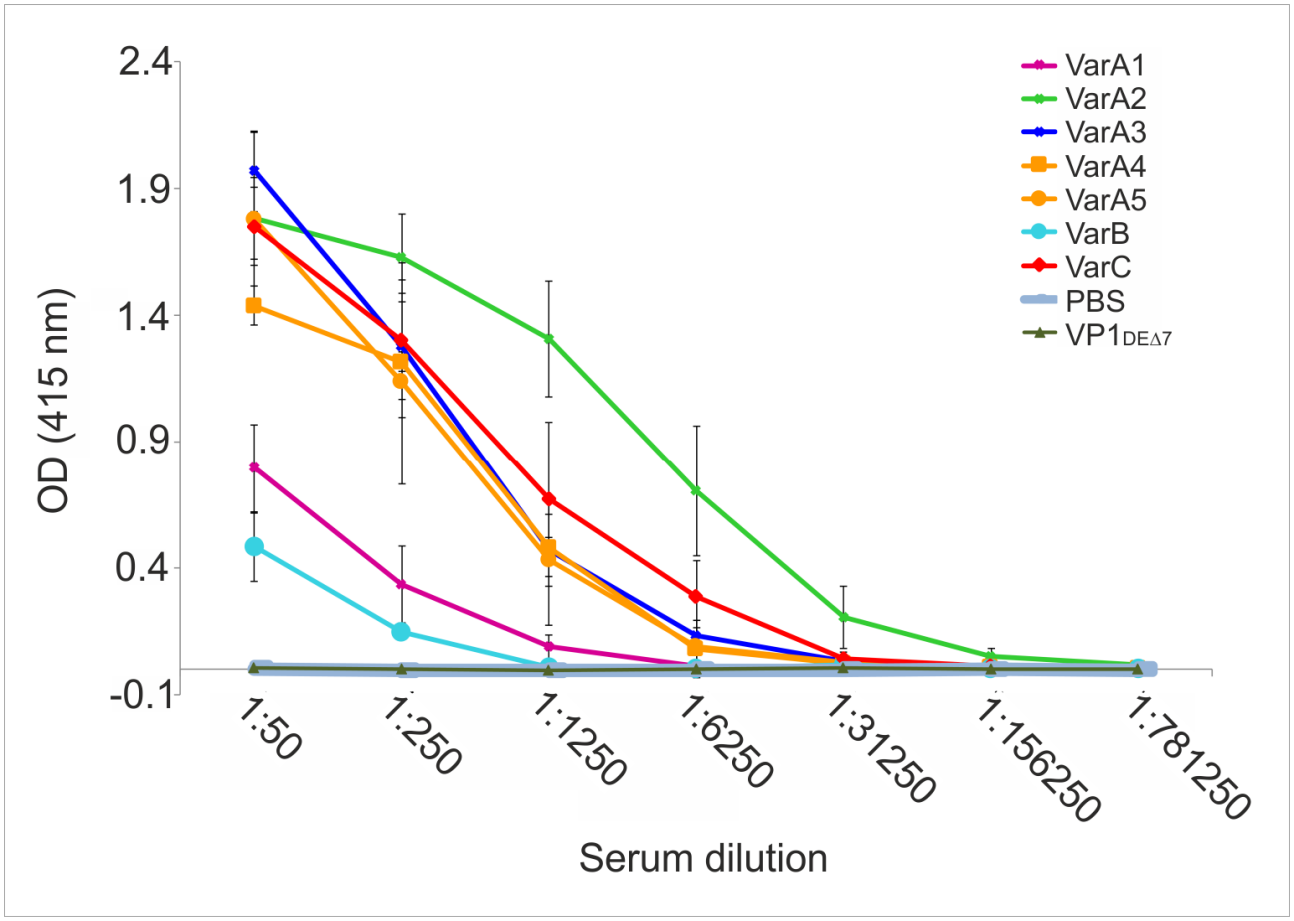
Cap-specific antibody response induced by candidate vaccine structures in mice. Groups of mice were immunized three times with individual candidate vaccine structures (50 μg/dose) or with PBS or VLPs composed of VP1_DEΔ7_ controls. The presence of specific antiPCV2 antibodies was examined using ELISA. Concentrated lysate of PCV2-infected PK15 cells was used as coating material.

Mice (five per group) were immunized with individual structures as described in Materials and Methods. Their humoral responses were tested by ELISA (using PCV2 virus as antigen) 14 days after the last immunization. In accordance with our previous findings [12, 64], high titers of antibodies against the VP1 protein were detected in all immunized mice (S1 Fig). Importantly, all immunized mice (except controls) developed antibodies specific for the PCV2 virus (Fig 5).

While levels of antibodies obtained after immunization of mice by VarA3, VarA4, VarA5 and VarC did not differ significantly, higher levels of antibodies were detected after immunization by VarA2 (visible particularly in dilutions 1:1250 and 1:6250), pointing to high immunogenicity of this epitope. As expected, the lowest level of Cap-specific antibodies was induced by VarB VLPs, that carry the Cap protein inside, not exposing its epitopes on particle surface. Moreover, whereas stoichiometric ratio of VP1 molecules versus Cap sequence is 1:1 in all other prepared nanostructures, in VarB, approximately 14 molecules of Cap protein is carried by one VLP (composed of 360 VP1 molecules).

A relatively low antibody response was also induced with the Cap epitope carried by VarA1 VLPs. This epitope was identified by Lekcharoensuk et al [45]. However, another study [65] did not detect any reactivity against the epitope carried by VarA1 nanostructure by testing sera of PCV2 infected pigs with individual peptides covering entire Cap protein sequence. Also, set of monoclonal antibodies, generated by Shang et al [47] from mice immunized by recombinant PCV2 Cap protein and from mice infected by isolated virions, did not find any reactivity with a fragment covering this epitope. It is probable that the epitope carried by VarA1 is a part of a conformation epitope and, when lifted out of the context, the epitope is poorly immunogenic. Furthermore, we cannot exclude a possible interaction of MPyV VP1 sequences with VarA1 epitope. According to the Swiss model (https://swissmodel.expasy.org), all epitopes inserted together with flexible linkers to MPyV VLPs should not interact with VP1 molecules. However, evaluation of computational structure prediction methods based on homology modelling revealed that loops longer than six amino acid residues are modelled inaccurately [66].

**Fig 6:**
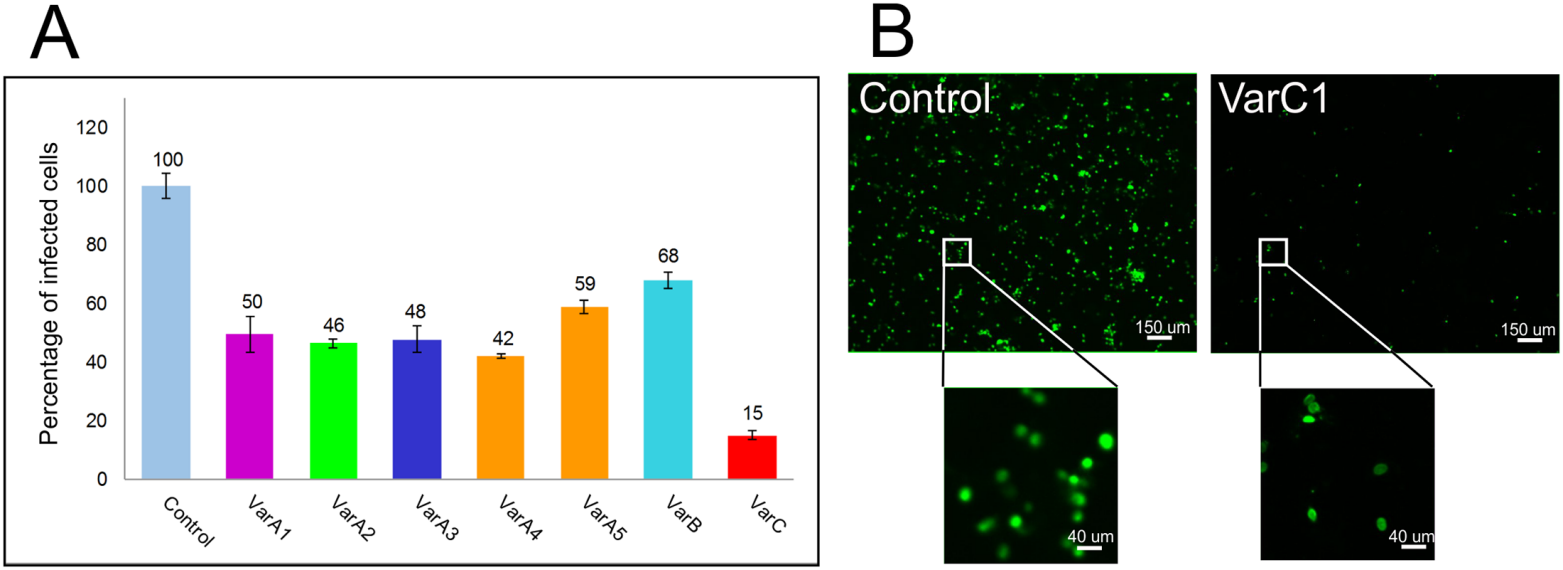
PCV2 virus neutralization by antibodies induced in mice. PK15 cells were inoculated with a mixture of 50-fold diluted sera collected from immunized pigs and PCV2 virus (final concentration TCID_50_ = 10^5^). Control cells were infected with the same amount of virus mixed with 50-fold diluted serum from mice immunized with PBS. Infected cells were detected 36 hpi by immunofluorescence assay with specific antiCap PCV2 antibody and the number of Cap PCV2-positive cells (green) was calculated. Columns represent percentage of infected cells relative to control. (**A**) Graph of neutralizing activity of mouse sera, (**B**) representative example of immunofluorescence staining of control cells and cells infected with virus incubated with sera of mice immunized by VarC1. Error bars represent standard error of three independent experiments. Bars represent 50 μm.

Next, we were interested in whether Cap-specific antibodies induced in mice by the prepared nanostructures could block PCV2 infection. PCV2 virus inoculum was mixed with individual inactivated mice sera and after 1 h incubation at 37°C, the mixtures were used for infection of PK15 cells. After 36 h, cells were fixed and the numbers of Cap-positive cells were counted. The sera containing Cap-specific antibodies were tested in the neutralization assay. Each serum mixture of mice immunized with VLPs carrying a Cap epitope (VarA) (in dilution 1:50) caused an approx. 50% decrease in infected cells compared to the control serum, reflecting the fact that all selected epitopes are part of a dominant conformation epitope on the capsid surface [49]. Introduction of a dimer of epitope 4 into VLPs (VarA5) did not significantly affect either antibody production or virus neutralization. The best results for virus neutralization were achieved after immunization with the VarC structure. The sera of mice immunized by VarC exhibited 85% neutralization activity at a dilution of 1:50 (Fig 6).

### VarC1 candidate vaccine induces cellular responses *in vitro*

**Fig 7:**
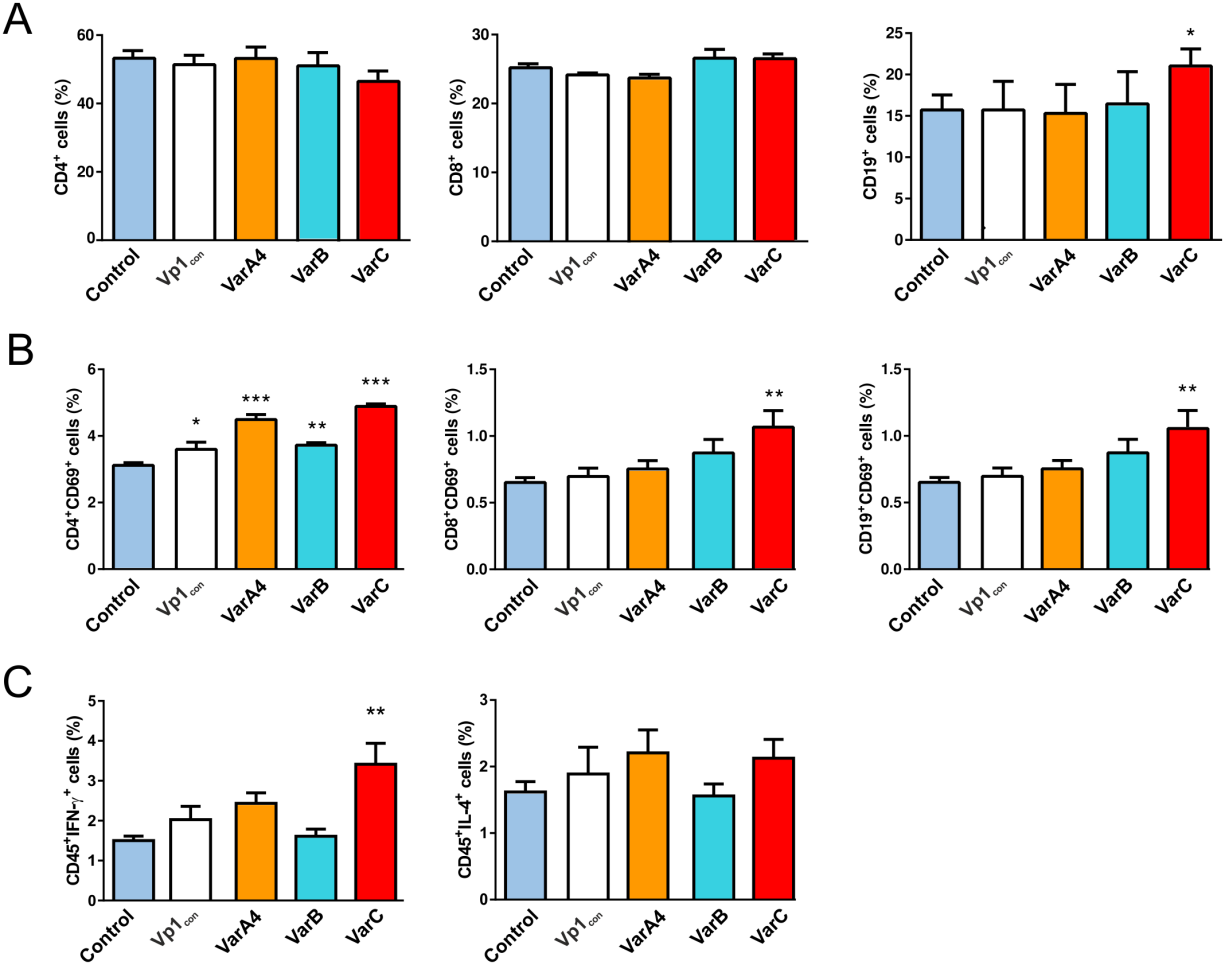
Cellular immune responses induced by candidate vaccines in mice tested *in vitro*. (**A**) Relative representation of Th CD4^+^, Tc CD8^+^ and B lymphocytes CD19^+^ in mouse splenocyte population after *in vitro* incubation with VarA4, VarB and VarC nanostructures. (**B**) Activation of Th CD4^+^, Tc CD8^+^ and B lymphocytes CD19^+^ in the mouse splenocyte population after *in vitro* incubation with VarA4, VarB and VarC nanostructures. (**C**) IFNγ and IL-4 intracellular level of mouse splenocyte CD45^+^ population after *in vitro* incubation with VarA4, VarB and VarC nanostructures. Blue and white columns represent controls: splenocytes without added nanostructures (control; blue) and MPyV VLPs without inserted PCV2 epitopes, scaffold only (VP1_Con_; white). Error bars represent standard error of the mean, SEM, *P <0.05, ** P<0.005, *** P < 0.0005.

Cell-mediated immunity is an important mechanism in the protection of organisms against viral infections. We performed pilot experiments to elucidate whether the prepared nanostructures were able to modulate the cellular immune response. Therefore, the representative of VarA nanostructures, VarA4, as well as VarB and VarC were tested in *in vitro* assays for changes in the population of leukocytes, their activation and cytokine production. In brief, spleen cells were cultured in the presence or absence of individual nanostructures and cell populations were phenotypically characterized by flow cytometry (Dot-plots and gating strategy of FACS analysis are in S2 Fig). As shown in Fig 7A, VarC alone significantly increased the proportion of CD19^+^ cells (Fig 7Ac), whereas populations of CD4^+^ (Fig 7Aa) and CD8^+^ (Fig 7Ab) cells remained unaltered by the presence of any tested candidate vaccines. This finding is consistent with the increased production of antibodies specific for the PCV2 virus.

Activation of T and B cell populations in the spleen cell culture by tested candidate vaccines was determined. As demonstrated in Fig 7B, all tested candidate vaccines activated CD4^+^ lymphocytes. A more apparent increase was observed when cells were cultured in the presence of VarA4 and VarC (Fig 7Ba). The frequency of CD8 (Fig 7Bb) and CD19 cells expressing the early activation marker CD69 (Fig 7Bc) was significantly increased only in the presence of VarC in the culture. The up-regulation of activation marker CD69 on a large proportion of T and B cells after acute viral infection has been documented [67]. As all lymphocyte populations contribute to the specific anti-viral response [68], it is important that the tested populations of T and B lymphocytes are activated by the candidate vaccine. CD69 has also been confirmed as a marker of the functionality of leukocytes after *in vitro* expansion [69].

To determine whether the candidate vaccines themselves may prime a Th1-polarized response in the splenocyte culture, we analyzed (by intracellular staining) the production of IFN-γ and IL-4 by CD45^+^ cells. A significant increase in the number of CD45^+^IFN-γ^+^ cells was detected in the presence of VarC in the culture (Fig 7Ca). As the number of CD45^+^IL-4^+^ cells did not show any decrease (Fig 7Cb), polarization of the culture in the Th-1 direction was not confirmed. A mixed Th1/Th2 response was also induced after immunization of BALB/c mice with chimeric VLPs prepared by insertion of a short epitope at four different sites in the yeast-expressed hamster polyomavirus major capsid protein VP1 [70] and in the mouse model of vaccination by a JEV (Japanese encephalitis virus) live-attenuated vaccine or recombinant modified vaccinia virus Ankara [71]. However, in both models, the proportion of Th1/Th2 population was established according to the immunoglobulin subclass distribution. Our results confirmed this finding using an *in vitro* assay. Altogether, increase of expression of activation markers of T-cells and simultaneous increase in CD45^+^IFNγ^+^ suggest that cellular response can be induced at least by VarC nanostructure. Further analyzes, including *in vivo* assays, will be required for verification and understanding of cellular response induction and the contribution of individual immune system components.

### VarC induces efficient production of antibodies including virus neutralization antibody against PCV2 in pigs

**Fig 8:**
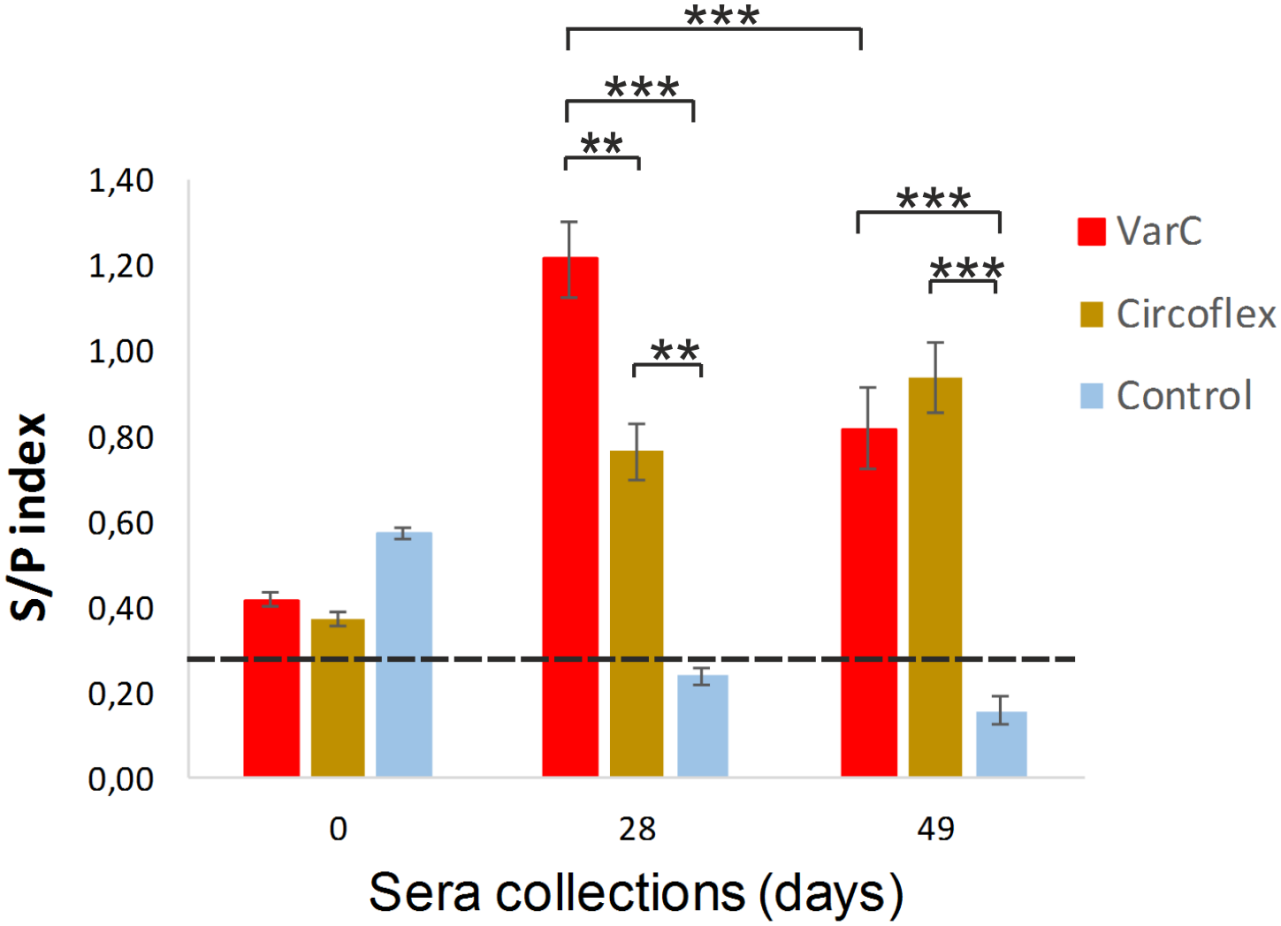
Comparison of PCV2-specific antibody responses induced by the VarC candidate vaccine to those induced by commercial Circoflex vaccine. Two groups of pigs were immunized twice (days 0 and 21) with nanostructure VarC or Circoflex vaccine according to the manufacturer’s instructions or with PBS containing 30% Polygen adjuvant. The levels of antiPCV2 antibodies were measured using a commercial ELISA kit (Ingezim Circo IgG) according to the manufacturer’s instructions. The S/P index—an index expressing the amount of antiPCV2-specific antibodies as a ratio between OD_415nm_ of the sample / OD_415nm_ of the control which is part of the ELISA kit. The dashed line represents the border of the S/P index (0.285) between sera that are negative and positive for PCV2-specific antibodies (calculated according to the ELISA kit manual). Error bars represent standard error of the mean, SEM, *P <0.05, ** P<0.005, *** P < 0.0005.

The candidate vaccine VarC, inducing antibodies in mice with the highest ability to neutralize the PCV2 virus, was selected for immunization of pigs. Prior to immunization, piglets (6 weeks old) were tested for the presence of PCV2 DNA in the blood by qPCR to reveal possible infection by PCV2. No virus DNA copies were detected. The animals were immunized as described in the Methods: one group (7 pigs) was immunized by VarC, one group of three pigs was immunized with the commercial Circoflex vaccine for comparison, and a control group (three pigs) was immunized using PBS and adjuvants only. The humoral response was tested using a commercial ELISA kit Ingezim Circo IgG. At the time of the first immunization (0 days), slightly elevated levels of antibodies specific for PCV2 were detected in all groups of animals (Fig 8). The levels appeared to represent the presence of maternal antibodies in the tested piglets. On day 28 after the first immunization, a significant rise in PCV2-specific antibodies was detected in the sera of pigs immunized with VarC or Circoflex vaccine, but not in the sera of control pigs. The level of PCV2-specific antibodies induced by VarC was significantly higher than that obtained by the Circoflex vaccine. However, 49 days after the first immunization (and 28 days after the second immunization), the level of antibodies induced by VarC decreased, so that the antibody levels induced by both experimental and commercial vaccines were statistically equal. We posit that the decrease in antibody level detected on the 49^th^ day post immunization by the VarC vaccine can be overcome by optimization of adjuvants. The levels of PCV2-specific antibodies of for non-immunized controls decreased over time to levels that are not considered positive serum.

**Fig 9:**
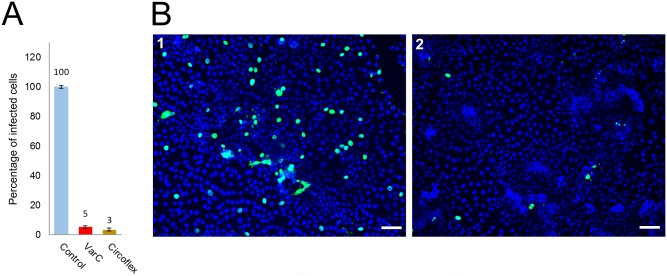
Pig serum neutralization assay. PK15 cells were inoculated with a mixture of 100-fold diluted sera collected from immunized pigs and PCV2 virus (final concentration TCID_50_ = 10^5^). Control cells were infected with the same amount of the virus mixed with 100-fold diluted serum from a gnotobiotic pig. Infected cells were detected 36 hpi by immunofluorescence assay with antibody against the Cap protein, and the numbers of PCV2-positive cells were calculated. (**A**) Plot of neutralizing activity of pig sera. The plots represent the percentage of infected cells relative to the control. Cells from 17 optical fields (approx. 1500 infected cells for the control sample) were counted in each experiment. Error bars represent standard error of three independent experiments. (**B**) Representative fields of counted cells. Control cells (**1**) and cells infected with the virus neutralized by VarC-induced antibodies (**2**). Blue—DAPI, green—PCV2 Cap protein. Bars represent 50 μm.

For the viral the clearance and recovery of pigs from infection, the presence of neutralizing antibodies is crucial. With the development of PMWS, the absence or impaired production of PCV2-specific neutralizing antibodies was observed [61, 72]. A decrease in viremia coincides with an increase in neutralizing antibody titers in infected pigs inoculated with PCV2 [72]. Therefore, we further investigated whether Cap-specific antibodies induced in pigs can block PCV2 infection. The PCV2 virus inoculum was mixed with different concentrations of inactivated pig sera (collected 28 days after the first immunization); after 1 h incubation at 37°C, PK15 pig cells were infected. Cells were fixed 36 hpi and stained for indirect immunofluorescence. The numbers of Cap-positive cells were counted. The results obtained with 100-fold diluted sera are presented in Fig 9. Comparable virus neutralization results were obtained for both VarC and Circoflex vaccines.

The first commercial PCV2 vaccine (Circovac, Merial), introduced in 2006, was based on an inactivated oil-adjuvanted vaccine. The Cap protein of PCV2a expressed in the baculovirus system is the antigen of three other vaccines (Circoflex, Boehringer Ingelheim; Circumvent, Intervet/Merck; Porcillis PCV, Schering-Plough/Merck). Two other vaccines, Suvaxyn (Fort Dodge Animal Health) and Fostera PCV (Pfizer Animal Health) are based on a chimeric PCV1/ 2 virus containing the genomic backbone of the non-pathogenic PCV1, with the cap gene replaced by that of PCV2. All these vaccines are directed against the PCV2a subtype. Although an improvement in the expression of self-contained Cap protein in insect cells has been achieved [73,74], in our hands, the production and isolation of VarC chimeric structures carrying the fused Cap protein of PCV2b subtype was more efficient and easy. Another advantage of the VarC chimeric vaccine is that it represents a so-called DIVA (differentiating infected from vaccinated animals) vaccine, which also induces an immune response that differs from the response induced by natural infection, in this case, antibodies against the mouse polyoma VP1 protein.

## Supporting information

S1 File PCV2 Cap DNA and amino acid sequences. (DOCX)

S1 Table Primers used in this study. (DOCX)

S2 Table Phosphorylated oligonucleotides used in this study. (DOCX)

S1 Fig Antibody response against the VP1 protein in immunized mice. (TIF)

S2 Fig Dot-plots and gating strategy of FACS analysis. (PDF)
