# Supplementary material for: Exploitation of stable nanostructures based on the mouse polyomavirus for development of a recombinant vaccine against porcine circovirus 2
Source: PLoS One. 2017 Sep 18;12(9):e0184870. doi: 10.1371/journal.pone.0184870 (PMC5602543; doi:10.1371/journal.pone.0184870)
Supplement: S1 File — (DOCX) [file pone.0184870.s001.docx]

# S1 sequences

PCV2 Cap DNA sequence

ATGACGTATCCAAGGAGGCGTTACCGGAGAAGAAGACACCGCCCCCGCAGCCATCTTGGCCAGATCCTCCGCCGCCGCCCCTGGCTCGTCCACCCCCGCCACCGTTACCGCTGGAGAAGGAAAAATGGCATCTTCAACACCCGCCTG**TCCCGCACCTTCGGATATACTGTCAAGCGAACCACAGTCAGAACGCCCTCC**TGGGCG**GTGGACATGATGAGATTCAATATTAATGACTTTCTTCCCCCA**GGAGGGGGCTCAAACCCCCGCTCTGTGCCCTTTGAATACTACAGAATAAGAAAGGTTAAGGTTGAATTCTGGCCCTGCTCCCCGATCACCCAGGGTGACAGGGGAGTGGGCTCAAGTGCTGTTATTCTAGATGATAACTTTGTAACAAAGGCCACAGCCCTCACCTATGACCCCTATGTAAACTACTCCTCCCGCCATACCATAACCCAGCCCTTCTCCTACCACTCCCGCTACTTTACCCCCAAACCTGTCCTAGATTCCACTATTGATTACTTCCAACCAAACAACAAAAGAAACCAGCTGTGGC**TGAGACTACAAACTGCTGGAAATGTAGACCACGTAGGCCTCGGCACTGCGTTC**GAAAACAGTATATACGACCAGGAATACAATATCCGTGTAACCATGTATGTACAATTCAGAGAA**TTTAATCTTAAAGACCCCCCACTTAACCCT**TAA

PCV2 Cap amino acid sequence

MTYPRRRYRRRRHRPRSHLGQILRRRPWLVHPRHRYRWRRKNGIFNTRL**SRTFGYTVKRTTVRTPS**WA**VDMMRFNINDFLPP**GGGSNPRSVPFEYYRIRKVKVEFWPCSPITQGDRGVGSSAVILDDNFVTKATALTYDPYVNYSSRHTITQPFSYHSRYFTPKPVLDSTIDYFQPNNKRNQLW**LRLQTAGNVDHVGLGTAF**ENSIYDQEYNIRVTMYVQFRE**FNLKDPPLNP***
