# Supplementary material for: Exploitation of stable nanostructures based on the mouse polyomavirus for development of a recombinant vaccine against porcine circovirus 2
Source: PLoS One. 2017 Sep 18;12(9):e0184870. doi: 10.1371/journal.pone.0184870 (PMC5602543; doi:10.1371/journal.pone.0184870)
Supplement: S1 Table — (DOCX) [file pone.0184870.s002.docx]

# S1_table

| Primers (5´-3´) | |
| --- | --- |
| VP1a EcoRI | GATGATGAATTCATGGCCCCCAAAAGAAAAAGCGGC |
| VP1a BamHI | GATGATGGATCCTGAGCCGCTACCGGGTTTGTTGAACCCATG |
| VP1b BamHI | GATGATGGATCCGGGTCTGGCTCTGGAATTTCCACTCCAGTG |
| VP1b KpnI | GCTGCTGGTACCTTAATTTCCAGGAAATACAGTCTTTG |
| FLAGCap SmaI | CTCGTACCCGGGATGGATTACAAGGACGATGACGACAAGACGTATCCAAGGAGGC |
| Cap SacI | CTCGTAGAGCTCTGAGGTTAAGTGGGGGGTC |
| VP1 BglII BamHI | CTCGTAAGATCTGGATCCATGGCCCCCAAAAGAAAAAG |
| VP1 SalI | GAAGTTGTCGACATTTCCAGGAAATACAGTCTTTG |
| Cap BamHI | GAAGTTGGATCCATGACGTATCCAAGGAGGC |
| CapHis KpnI | CTCGTAGGTACCTCAGTGGTGGTGGTGGTG |
