# Supplementary material for: Exploitation of stable nanostructures based on the mouse polyomavirus for development of a recombinant vaccine against porcine circovirus 2
Source: PLoS One. 2017 Sep 18;12(9):e0184870. doi: 10.1371/journal.pone.0184870 (PMC5602543; doi:10.1371/journal.pone.0184870)
Supplement: S2 Table — (DOCX) [file pone.0184870.s003.docx]

# S2_table

| Phosphorylated oligonucleotides (5´-3´) | |
| --- | --- |
| VarA1 FW | GATCCTCCCGCACCTTCGGATATACTGTCAAGCGAACCACAGTCAGAACGCCCTCCG |
| VarA1 Rev | GATCCGGAGGGCGTTCTGACTGTGGTTCGCTTGACAGTATATCCGAAGGTGCGGGAG |
| VarA2 FW | GATCCGTGGACATGATGAGATTCAATATTAATGACTTTCTTCCCCCAG |
| VarA2 Rev | GATCCTGGGGGAAGAAAGTCATTAATATTGAATCTCATCATGTCCACG |
| VarA3 FW | GATCCCTGAGACTACAAACTGCTGGAAATGTAGACCACGTAGGCCTCGGCACTGCGTTCG |
| VarA3 Rev | GATCCGAACGCAGTGCCGAGGCCTACGTGGTCTACATTTCCAGCAGTTTGTAGTCTCAGG |
| VarA4 (A5) FW | GATCCGGTTCTGGTTCTTTTAATCTTAAAGACCCCCCACTTAACCCTGGTTCTGGTTCTG |
| VarA4 (A5) Rev | GATCCAGAACCAGAACCAGGGTTAAGTGGGGGGTCTTTAAGATTAAAAGAACCAGAACCG |
