# Supplementary figures and images for: Exploitation of stable nanostructures based on the mouse polyomavirus for development of a recombinant vaccine against porcine circovirus 2
Source: PLoS One. 2017 Sep 18;12(9):e0184870. doi: 10.1371/journal.pone.0184870 (PMC5602543; doi:10.1371/journal.pone.0184870)

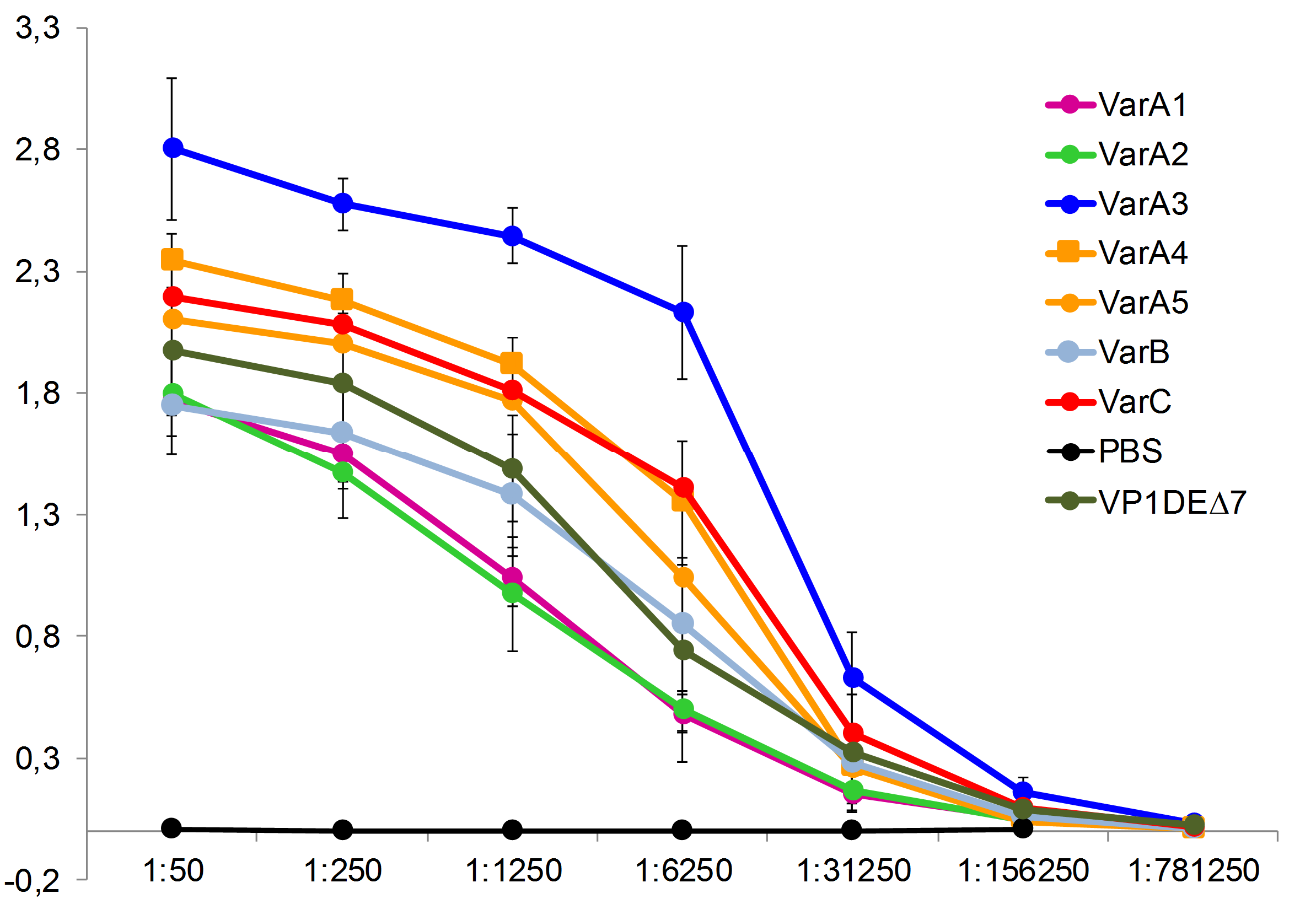

Supplement: S1 Fig — (TIF) [file pone.0184870.s004.tif]
