# Supplementary material for: Exploitation of stable nanostructures based on the mouse polyomavirus for development of a recombinant vaccine against porcine circovirus 2
Source: PLoS One. 2017 Sep 18;12(9):e0184870. doi: 10.1371/journal.pone.0184870 (PMC5602543; doi:10.1371/journal.pone.0184870)

FACS analyzes of cell immune response

CD4+ cells
Gating strategy

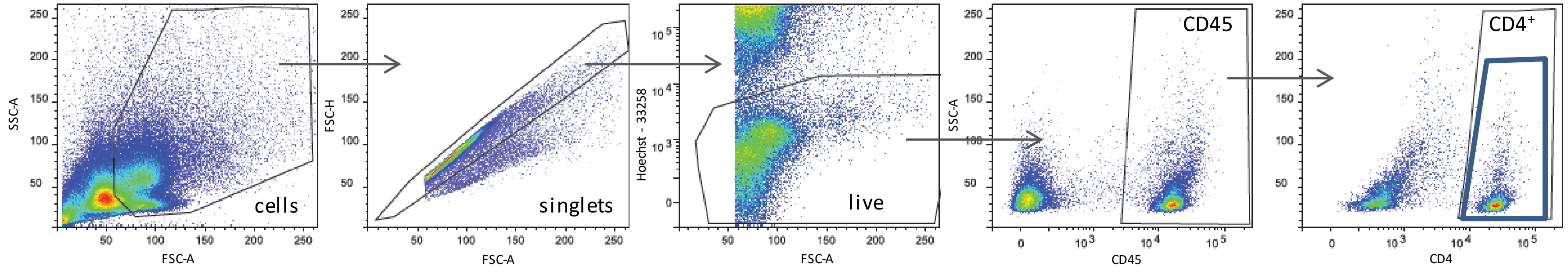

Representative dotplots

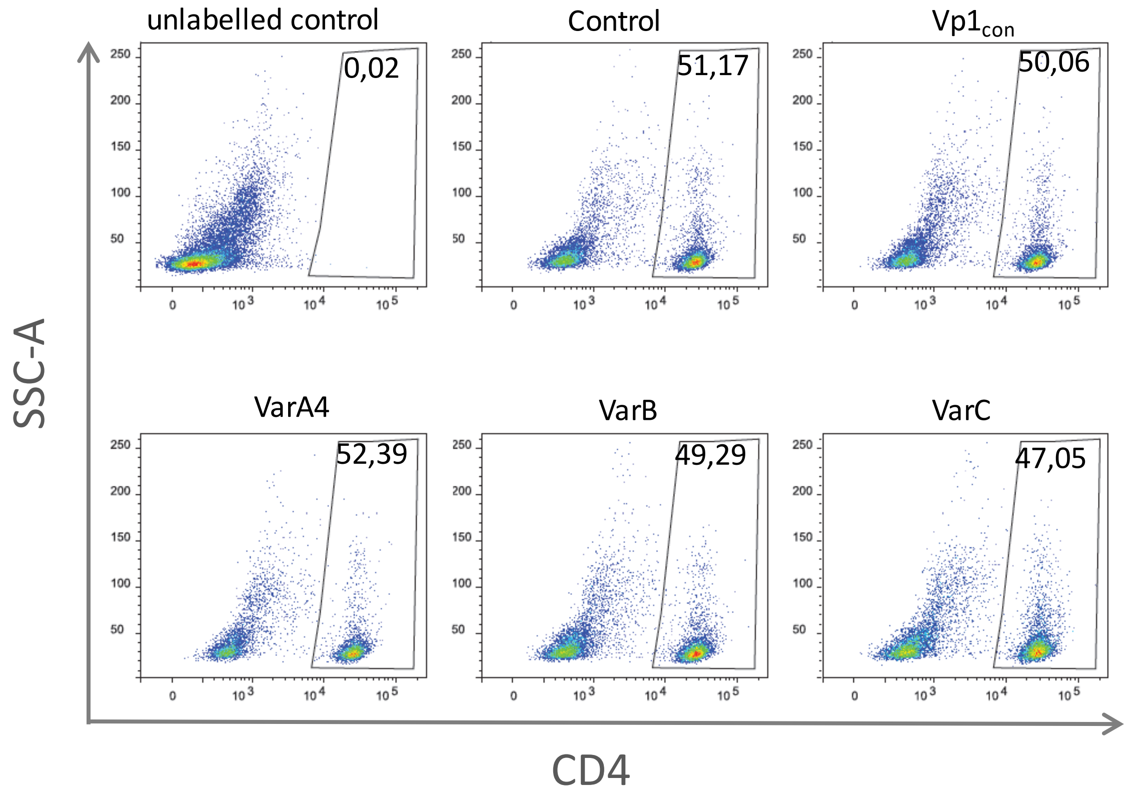

CD4$^+$CD69$^+$ cells
Gating strategy

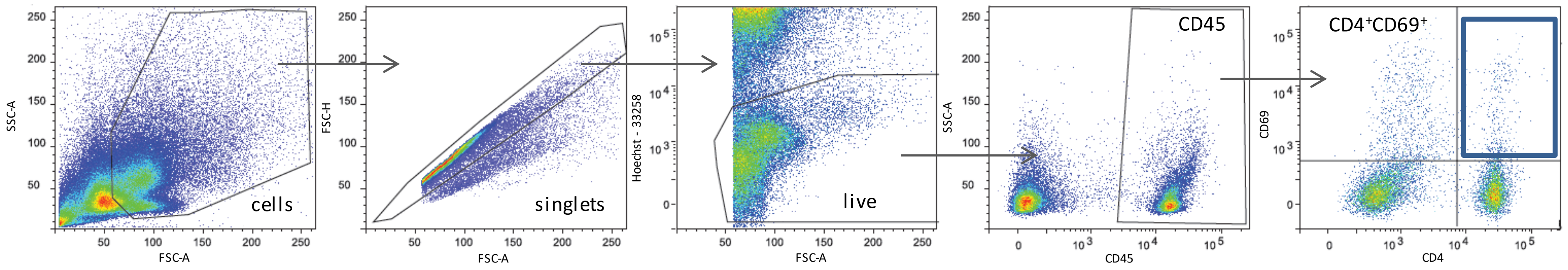

Representative dotplots

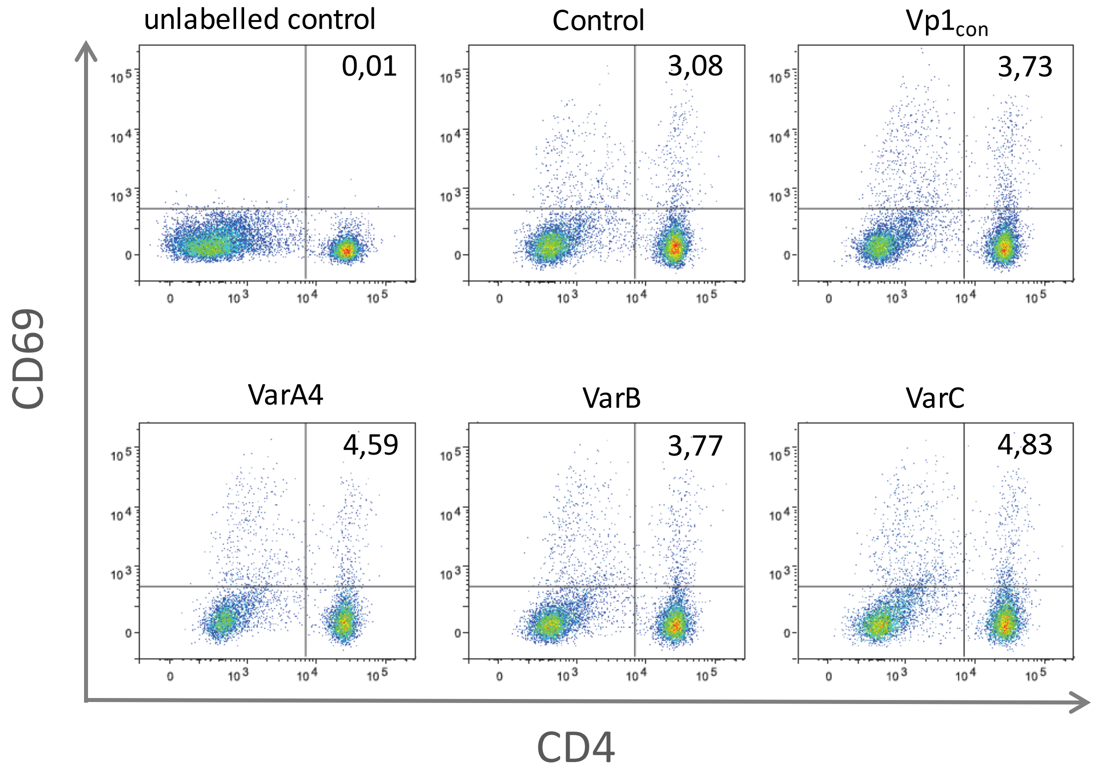

CD8+ cells
Gating strategy

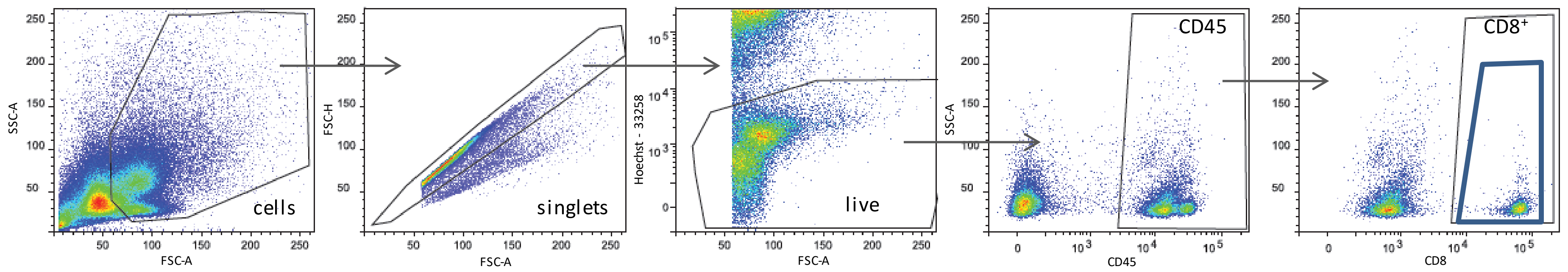

Representative dotplots

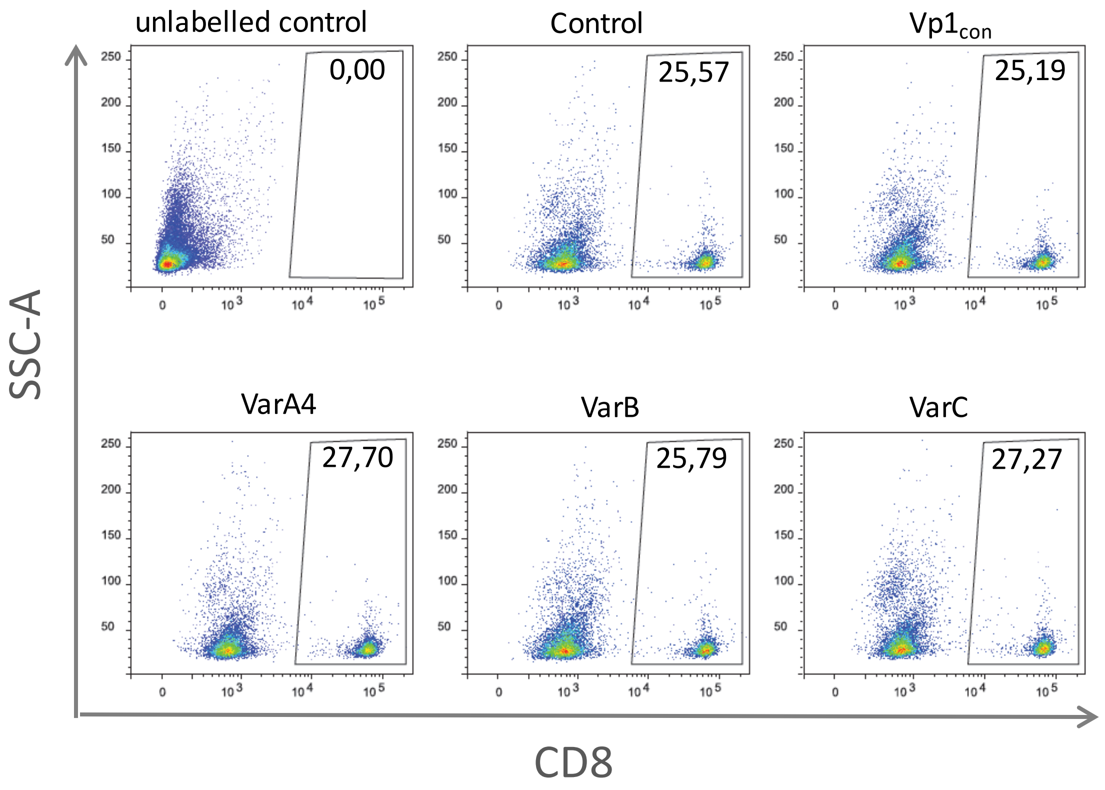

$CD8^+CD69^+$ cells
Gating strategy

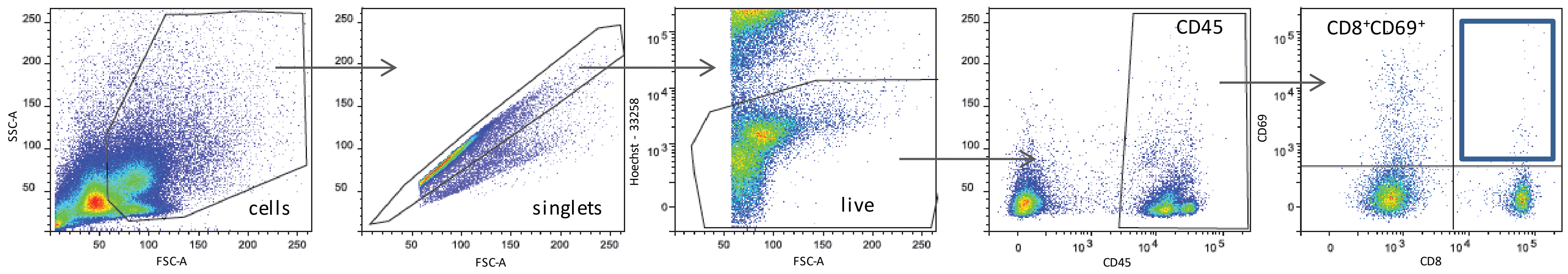

Representative dotplots

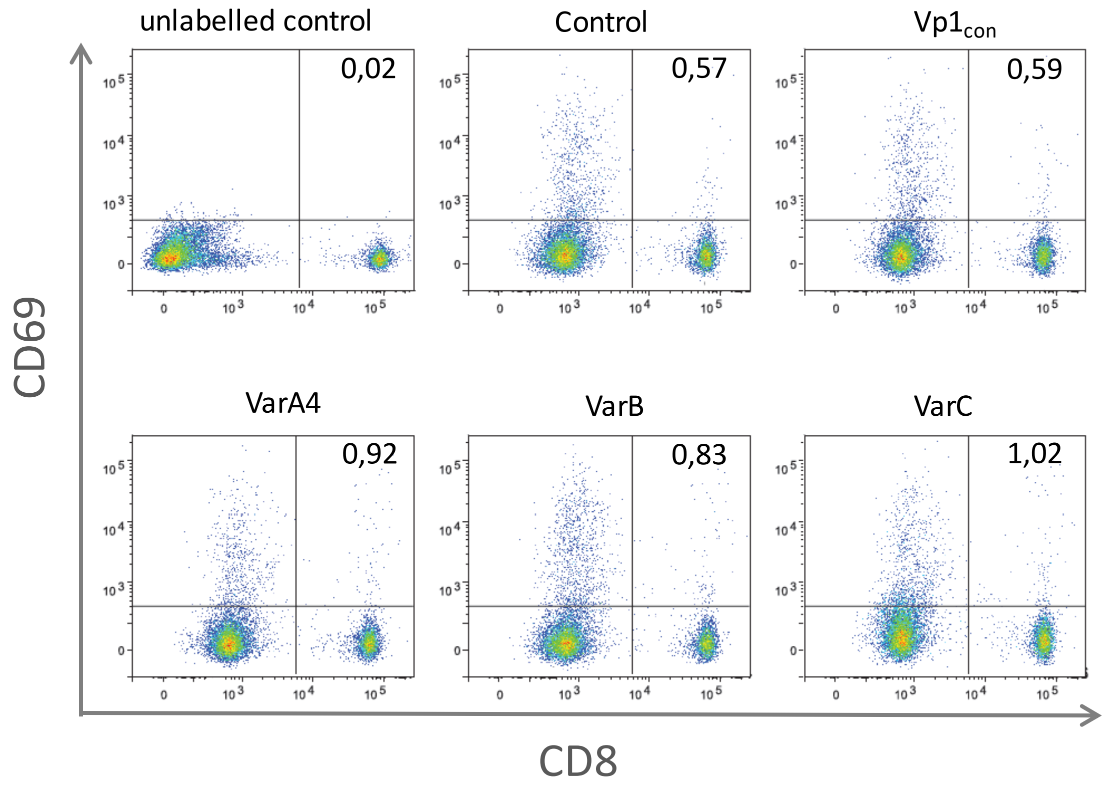

CD19+ cells
Gating strategy

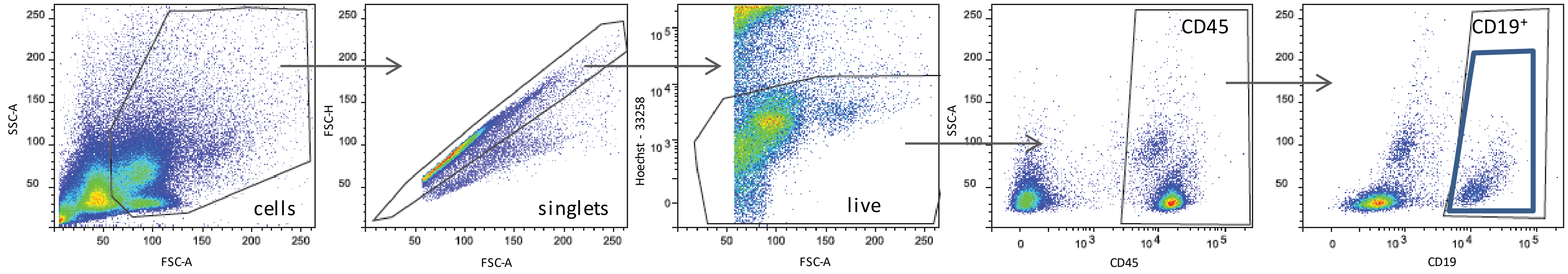

Representative dotplots

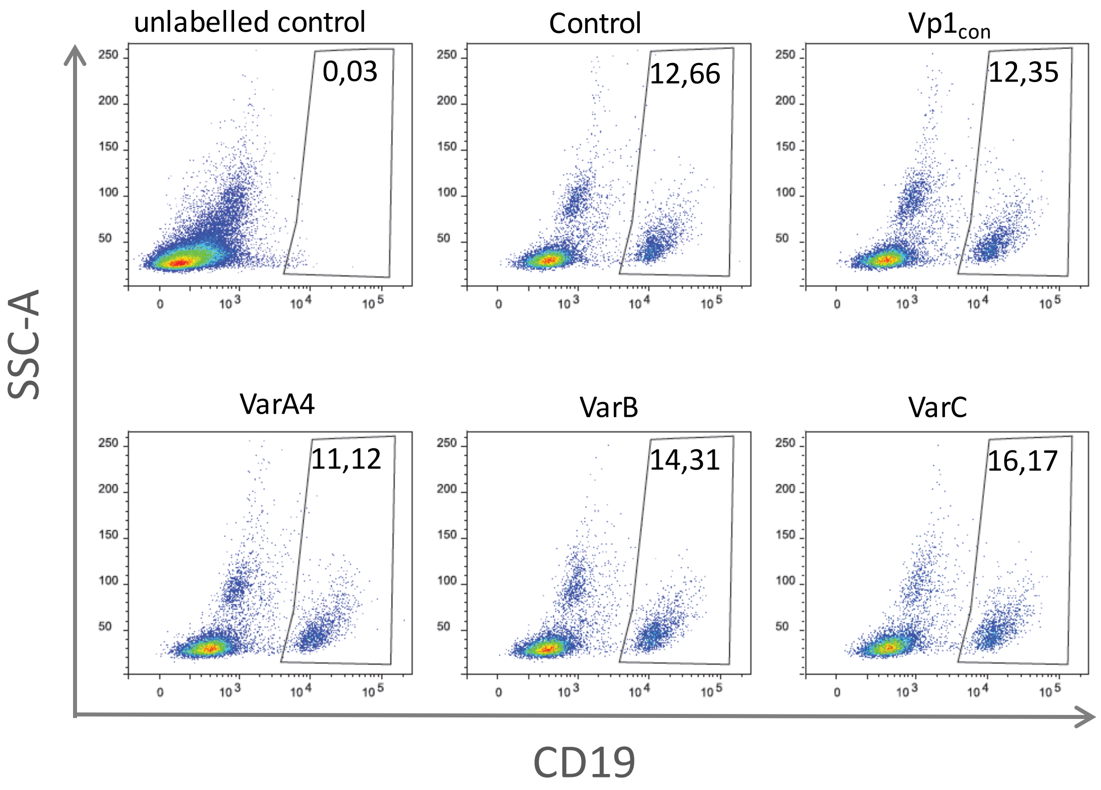

$CD19^+CD69^+$ cells
Gating strategy

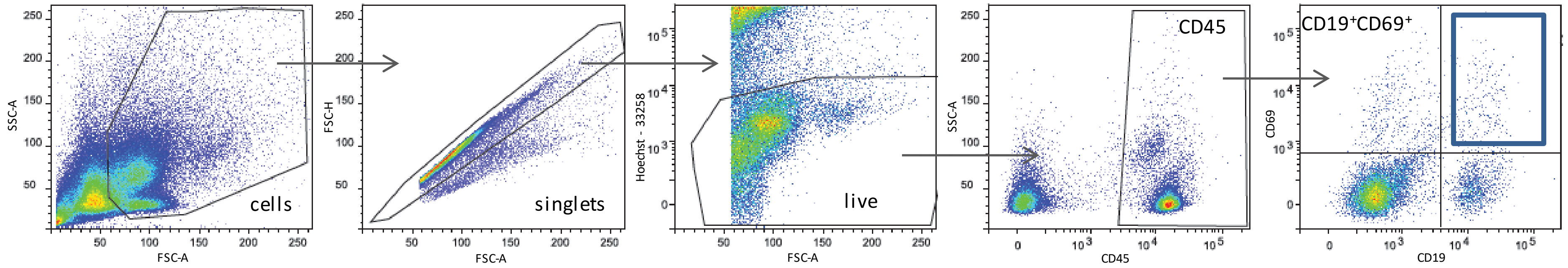

Representative dotplots

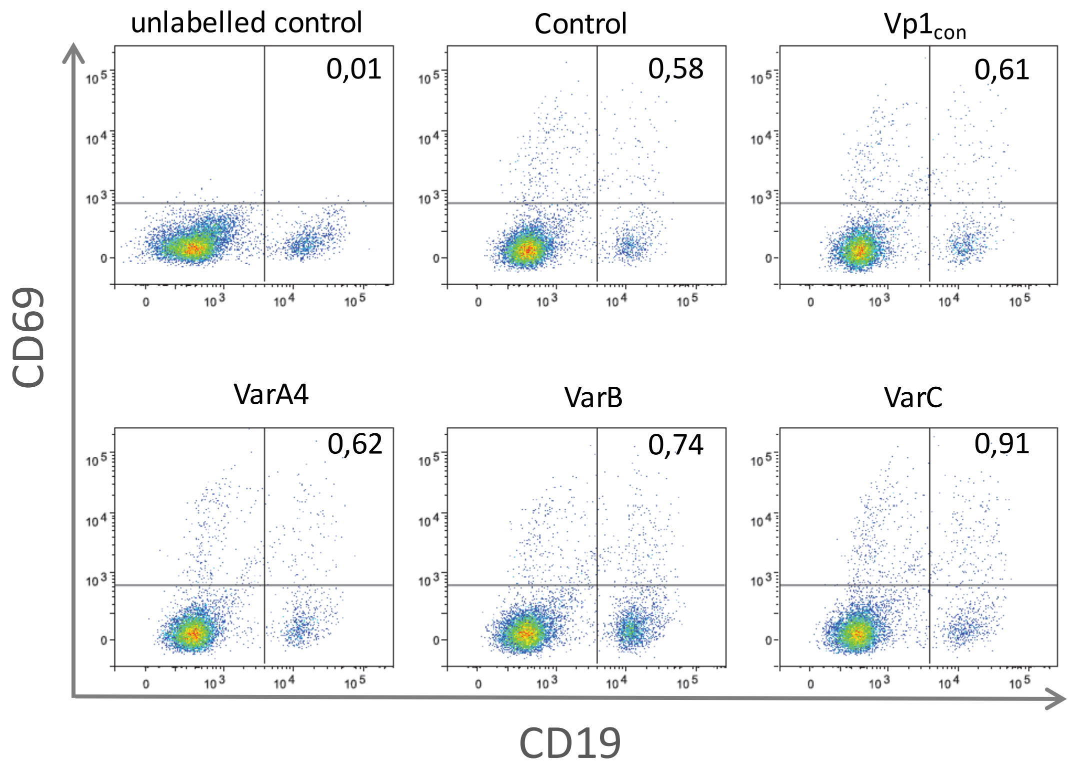

CD45⁺IL-4⁺ cells

Gating strategy

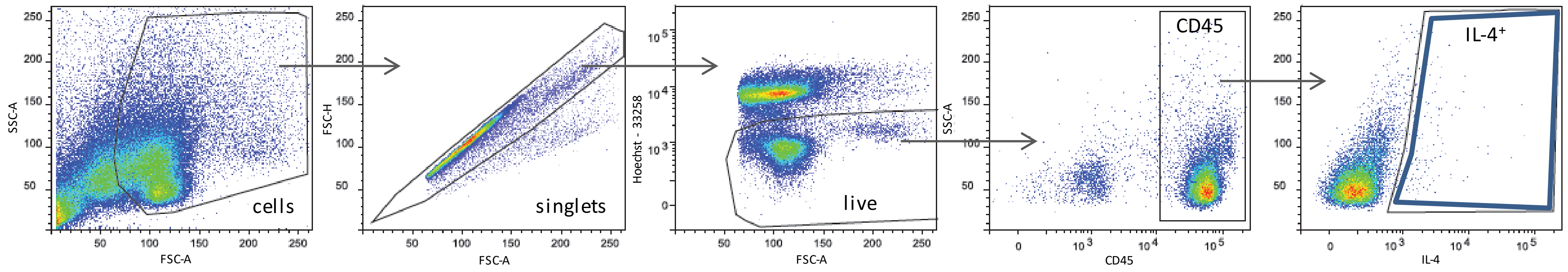

Representative dotplots

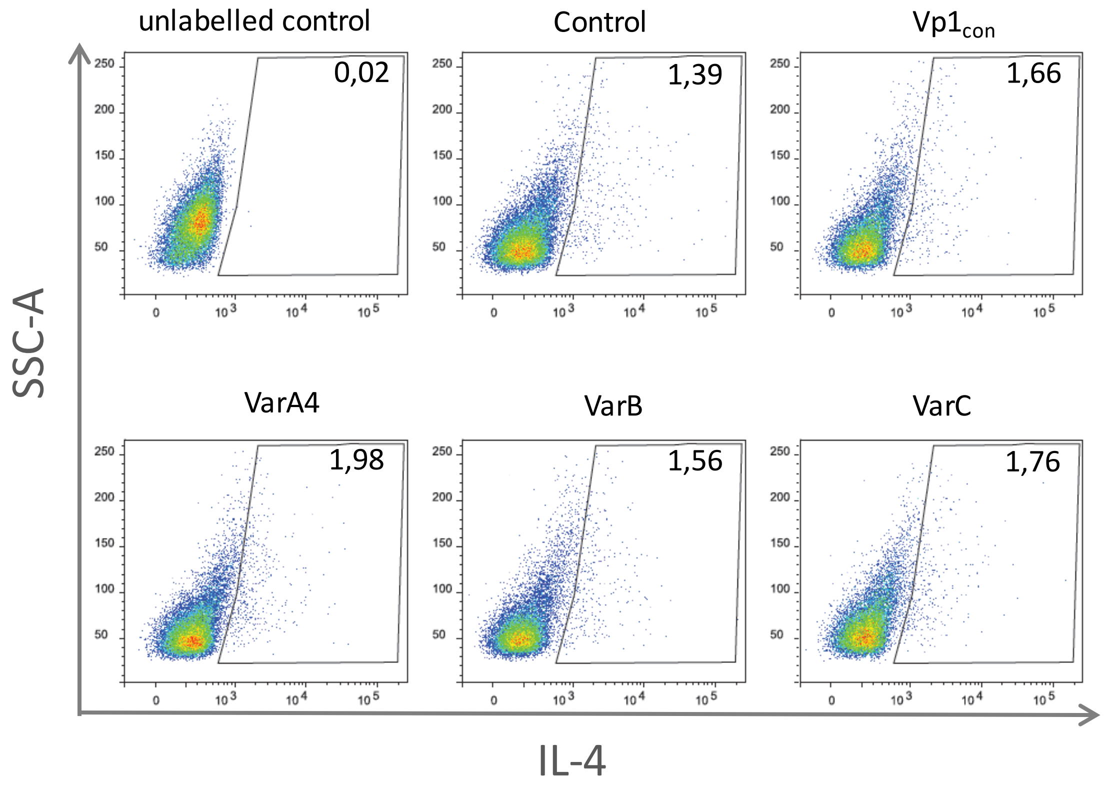

CD45$^+$IFN-γ$^+$ cells
Gating strategy

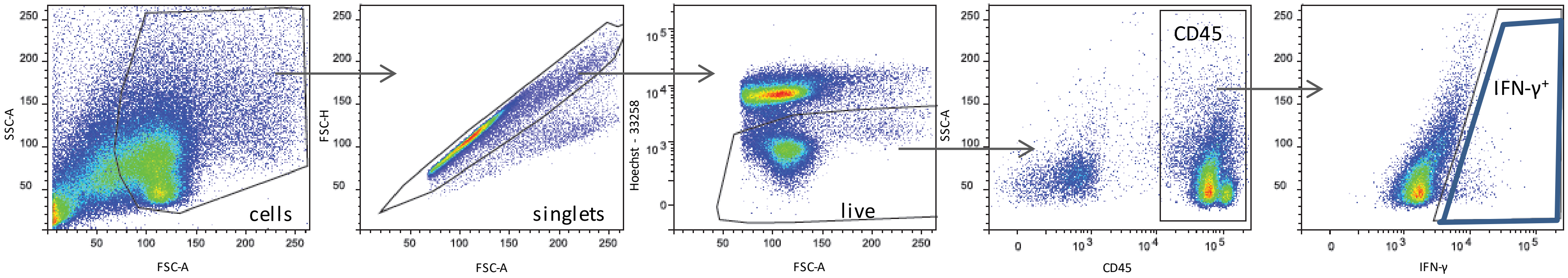

Representative dotplots

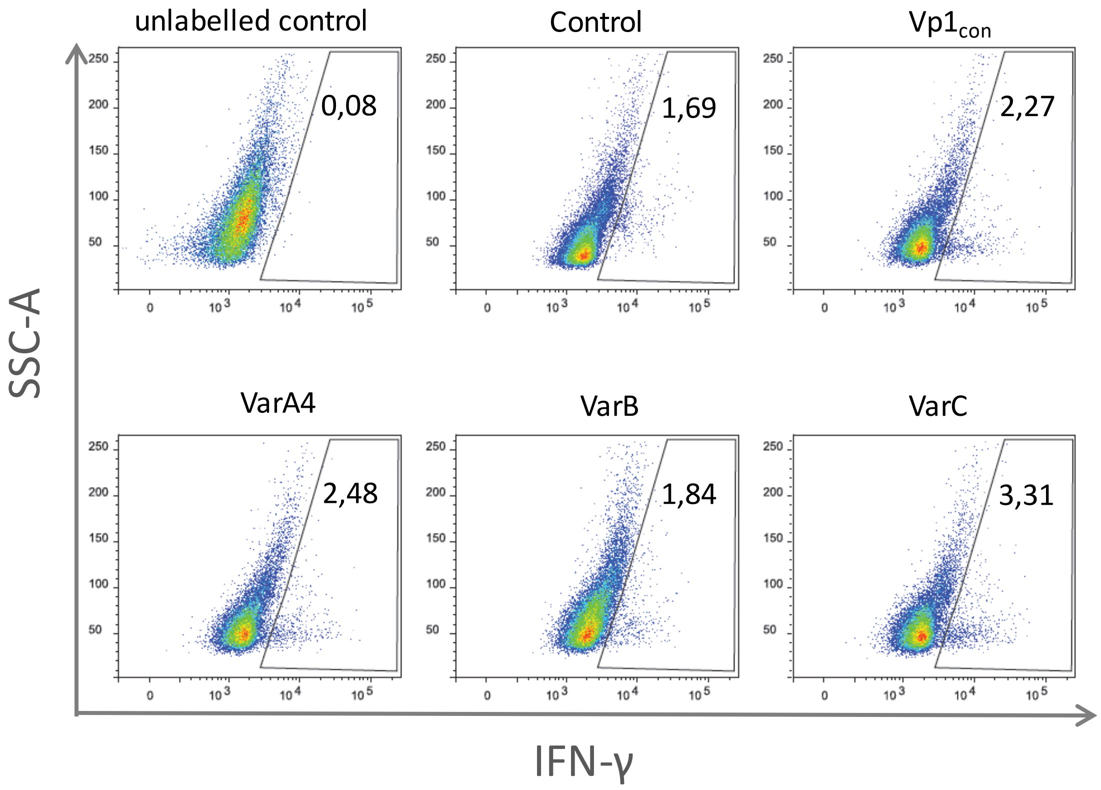

Supplement: S2 Fig — (PDF) [file pone.0184870.s005.pdf]
